# Risk of severe COVID-19 infection in persons with diabetes during the first and second waves in Denmark: A nationwide cohort study

**DOI:** 10.3389/fendo.2022.1025699

**Published:** 2022-10-11

**Authors:** Jacob V. Stidsen, Anders Green, Louise Rosengaard, Kurt Højlund

**Affiliations:** ^1^ Steno Diabetes Center Odense, Odense University Hospital, Odense, Denmark; ^2^ Open Patient data Explorative Network, Odense University Hospital, Odense, Denmark; ^3^ Department of Clinical Research, University of Southern Denmark, Odense, Denmark

**Keywords:** COVID-19, diabetes, cardiovascular disease, microvascular disease, hypertension, SARS-CoV-2, obesity, hospitalization

## Abstract

**Objective:**

Coronavirus disease-2019 (COVID-19) increases risk of hospitalization and death in diabetes and diabetes-related conditions. We examined the temporal trends in COVID-19-related hospitalization and mortality in the total Danish population by diabetes and diabetes-related conditions in the two first waves of COVID-19 in Denmark.

**Materials and methods:**

We identified all persons with diabetes in the whole Danish population using national registries. COVID-19-related risks of hospitalization and death were assessed using Cox regression analysis in wave 1 (1 March-31 August 2020) and wave 2 (1 September 2020-28 February 2021) of the pandemic for persons with (n=321,933) and without diabetes (n=5,479,755). Analyses were stratified according to status of hypertension, obesity, cardiovascular and microvascular disease.

**Results:**

The cumulative incidence of COVID-19 hospitalization increased from wave 1 to wave 2 in both persons without (from 4 to 10 in 10,000) and with diabetes (from 16 to 54 per 10,000). The relative risk of hospitalization, however, increased more in patients with diabetes compared to persons without (age-, sex- and co-morbidity-adjusted HR [aHR] 1.40 (95% CI 1.27, 1.55) versus 1.76 (1.65, 1.87), p<0.001 for interaction with wave). The mortality rate, according to the whole population, increased similarly in persons without and with diabetes from wave 1 to wave 2 (from 0.63 to 1.5 versus from 4.3 to 10 in 10,000; aHR 1.65; 1.34, 2.03 and 1.64; 1.43, 1.88). However, when mortality was restricted to the hospitalized population, the crude mortality fell from 26.8% to 19.6% in persons with diabetes, while only a minor decrease was seen in persons without diabetes (from 16.7% to 15.5%).

**Conclusion:**

The risk of COVID-19-related hospitalization increased more in persons with than without diabetes from wave 1 to wave 2 of the COVID-19 pandemic in the Danish population. However, mortality according to the whole population did not change, due to reduced mortality among hospitalized persons with diabetes.

## Introduction

Infection with severe acute respiratory syndrome coronavirus 2 (SARS-CoV-2) causes severe pneumonia as well as other severe extrapulmonary conditions (1). By 8.June 2022 more than 6.3 mio deaths of coronavirus disease 2019 (COVID-19) had been reported globally (https://covid19.who.int/). Early in the pandemic it was established that high age and various co-morbidities conferred increased risk of hospitalization, treatment at an intensive care unit (ICU) and death due to COVID-19 (2, 3). Especially diabetes has been established as a strong risk factor for severe COVID-19 infections (4, 5), but also conditions overrepresented in individuals with diabetes, such as obesity (6), established cardiovascular disease (7) and hypertension (8), have been reported as risk factors.

Measures to protect the population against COVID-19 related hospitalization and adverse outcomes of COVID-19 involve restrictions on societal activity (9, 10), protective guidelines in general (11), and advances in treatment (12). Shielding of high risk population groups has been part of the protective strategy in some countries, including Denmark, but the effect of such measures are uncertain (13–17). Moreover, acceptance and implementation of these measures change over time (18). However, to our knowledge the combined effect of these factors on the risk of hospitalization with COVID-19 and death in persons with diabetes between the first two waves has not been established. We hypothesized that the risk of adverse COVID-19 outcomes in persons with diabetes would change during the first two waves of the pandemic in Denmark. The subsequent emergence of COVID-19 vaccines has decreased the risk (19), and competes with the effect of other protective measures.

Therefore, we investigated and compared the risk of hospitalization, need for intensive care and related death in the Danish population with and without diabetes in the first and second wave of the pandemic, before the introduction of COVID-19 vaccination. As shielding guidelines for specific groups, e.g. persons with diabetes, are dependent on precise risk estimation, we furthermore examined the combined effect of diabetes status and diabetes-related conditions such as hypertension, obesity, cardiovascular disease and microvascular disease on the risk of COVID-19 related outcomes.

## Materials and methods

### Population and data sources

The Danish Civil Registration System includes data on citizenship, date of death (if any) and migration status for all Danish citizens (20). Using the unique civil registration number assigned to all Danish citizens, person-level linkage can be made between the content of all national health registries. All citizens of Denmark alive and not migrated by March 1^st^, 2020 were eligible for analysis of the first wave and by September 1^st^, 2020 for analysis of the second wave of the COVID-19 pandemic. Diagnosis codes from all hospital contacts, as well as codes for diagnostic and medical procedures and surgical interventions were obtained from the Danish National Patient Registry (DNPR) that has recorded all hospital contacts in Denmark since 1977 (outpatient contacts since 1995) (21). All medications redeemed at Danish community pharmacies were available from the National Prescription Registry (NPR) that has been in operation since 1995 (22). Results from Polymerase Chain Replication (PCR) tests for SARS-CoV-2 were obtained from The Danish Microbiology Database (MIBA) (23).

### Outcomes

The primary outcomes were COVID-19 related hospitalization and COVID-19 related mortality. COVID-19 related death was defined as death within 30 days of the start of a COVID-19 related hospitalization. COVID-19 related hospitalization was defined as hospitalization for more than 12 hours within 30 days prior and 14 days after a positive test for SARS-CoV-2 and with a recorded COVID-19 diagnosis for the hospital contact. In case multiple hospitalizations in this period were present, the first contact was used. The date of the outcome was defined by the first day of COVID-19 related hospitalization. Secondary outcomes were COVID-19 related admission to ICU. COVID-19 related admission to ICU was defined as intensive unit care within 30 days of COVID-19 related hospitalization. Precise definitions are given in electronic supplementary material **(ESM) Table 1**.

### Variables

Diabetes was defined by any ICD-10 diagnosis codes of diabetes in DNPR or redemption of glucose-lowering medication in the whole time-span of the registries, except registrations coincident with a diagnosis of polycystic ovarian syndrome or gestational diabetes (**ESM Table 2**). Type 1 diabetes was defined by at least two redemptions of insulin, no redemption of non-insulin glucose-lowering treatment, and at least one ICD-10 diagnosis code for type 1 diabetes (**ESM Table 2**), while all others with diabetes were defined as having type 2 diabetes. Hypertension was defined by any ICD-10 diagnosis code for hypertension or redemption of any anti-hypertensive treatment in the whole timespan of the registries. Obesity was defined by ICD-10 diagnosis codes for obesity, operation codes for bariatric surgery or redemption of weight-lowering drugs (with other indication than diabetes). Cardiovascular disease was defined by diagnoses or operation codes defining ischemic heart disease, cerebrovascular disease, heart failure, peripheral and central ischemic disease and lower limb amputations. Microvascular disease was defined by diagnosis, procedure and operation codes defining chronic kidney disease, retinopathy or maculopathy and polyneuropathy. Other co-morbidity was defined by modified groups according to the Charlson Comorbidity Index (dementia, chronic pulmonary disease, connective tissue disease, ulcer disease, any liver disease, hemiplegia and cancer) as defined in **ESM Table 3**.

### Statistical analysis

The analyses were restricted to two separate periods: The first wave from 1.3.2020-31.8.2020 and the second wave from 1.9.2020-28.2.2021. The index date was defined as 1.3.2020 for the first wave and as 1.9.2020 for the second wave. The definition of the time periods of the waves were based on the following considerations: 1) The waves had to be comparable in length. 2) The time periods should include one period with a rapidly increasing hospitalization rate, including the peak. 3) The waves should be free of any significant use of vaccines. 4) Significant changes in the nature of the variants should be as low as possible. Overall, the difference between the waves were therefore the difference in the enforcement of general societal measures. For each wave we constructed cumulative incidence curves for each outcome, for individuals with and without diabetes, censoring non-COVID-19 mortality and emigration. The COVID-19 related mortality and risk of hospitalization were assessed in the total Danish population. As the risk of COVID-19 mortality, as defined in our study, is determined by two components: The risk of COVID-19 hospitalization in the overall population and the risk of death in the admitted population, we also calculated the mortality according to the admitted population to illustrate these pathways. We further calculated the corresponding 6-months cumulative incidences, overall and stratified by age and sex. We used cox regression analysis to estimate adjusted hazard ratios of the endpoints for the first and second wave. Persons were followed until an outcome event, death, emigration or end of study period, whichever came first. Furthermore, admitted individuals who did not experience the endpoint was censored after 30 days for the events death and ICU admission. Besides the unadjusted model we adjusted for age and sex in a second models and for the main non-stratified analysis we further adjusted for co-morbidity (cardiovascular disease, microvascular disease, obesity, hypertension, dementia, chronic pulmonary disease, connective tissue disease, ulcer disease, any liver disease, hemiplegia and cancer) in a fully adjusted model. We explored the interaction between diabetes status and wave. We then performed stratified cox regression analysis for selected diabetes-related conditions and treatments: age, sex, diabetes type, hypertension, obesity, cardiovascular disease, microvascular disease and lipid-lowering treatment adjusted for age and sex. Each diabetes-related condition was stratified by diabetes status, yielding 4 subgroups for each analysis with persons without both diabetes and the diabetes-related conditions as the reference. Stratification is only presented for the second wave as the infection was more uniformly distributed compared to the first wave. To elucidate differences in treatment success, Cox regression for ICU admission was also explored as a secondary outcome, with both the hospitalized population and the total population as a base. The risk of a COVID-19 infection was assessed as the first positive SARS-CoV-2 PCR test in a COX regression model, adjusted as described for the main analyses above. In two sensitivity analyses we tested the risk of COVID-19 related hospitalization within 14 days of the first positive test and the risk of death within 30 days of the first positive test. All analyses including first positive PCR test were done for each wave separately and therefore the interaction between wave and diabetes was not assessed.

The Cox proportional hazards assumption was assessed by plotting the natural log of the cumulative hazard function for those with and without diabetes in each wave, against the natural log of time and then checked for parallelism. The proportional hazard assumption was not fulfilled for the Cox regression analyses in analyses when both waves were in the same model as the cumulative incidence cross and for these analyses the HR should be interpreted as averages over the entire waves. All analyses were performed with STATA version 17.

## Results

In the total Danish population 321,933 had diabetes and 5,479,755 did not have diabetes at the start of the first wave. The prevalence of hypertension and obesity was higher among persons with diabetes as was diabetes-related complications, including cardiovascular and microvascular disease (**Table 1**).

**Table 1:** Basic characteristics of all persons with and without diabetes in Denmark.

	Persons with diabetes	Persons without diabetes
Total	321,933	5,479,755
Males, n (%)	170,374 (52.9%)	2,714,576 (49.5%)
Females, n (%)	151,559 (47.1%)	2,765,179 (50.5%)
0-69 years, n (%)	193,253 (60.0%)	4,768,399 (87.0%)
70-79 years, n (%)	85,587 (26.6%)	480,363 (8.8%)
80+ years, n (%)	43,093 (13.4%)	230,993 (4.2%)
Type 1 diabetes, n (%)	25,040 (7.8%)	
Type 2 diabetes, n (%)	296,893 (92.2%)	
Microvascular disease, n (%)	54,812 (17.0%)	66,845 (1.2%)
Cardiovascular disease, n (%)	82,018 (25.5%)	307,534 (5.6%)
Obesity, n (%)	63,490 (19.7%)	195,589 (3.6%)
Hypertension, n (%)	255,063 (79.2%)	1,295,980 (23.7%)

### Primary outcomes: Risk of hospitalization and mortality

Covid-19 related hospitalization was observed in a total of 2604 and 7347 persons in the first and second wave, respectively (**Table 2**). The crude 6-months cumulative incidence increased from wave 1 to 2 in persons without diabetes (from 4 to 10 in 10,000) and in persons with (from 16 to 54 in 10,000, **ESM Figure 1**, **ESM Table 4**). The risk of COVID-19 related hospitalization was higher in persons with diabetes in both waves after adjustment for age and sex (HR 2.33; 95% CI 2.11, 2.57 and 2.90; 95% CI 2.74, 3.07) and in the fully adjusted model (**Table 3**). Importantly, the relative risk in persons with diabetes compared to persons without diabetes increased from wave 1 to 2 (HR 1.40; 95% CI 1.27, 1.55 vs 1.76; 95% CI 1.65, 1.87; p<0.001 for interaction; **Table 3**).

**Table 2:** Number of persons with COVID-19 infection, COVID-19 related hospitalization, death and intensive unit care (ICU) admission in the first and second wave of the pandemic, stratified by diabetes status.

	Persons with diabetes		Persons without diabetes		Hospitalized persons with diabetes		Hospitalized persons without diabetes	
	Wave 1 n=321,933	Wave 2 n=325,555	Wave 1 n=5,479,755	Wave 2 n=5,429,502	Wave 1 n=519	Wave 2 n=1758	Wave 1 n=2085	Wave 2 n=5589
COVID-19 infection*	1389 (0.43%)	9896 (3.05%)	15503 (0.28%)	181705 (3.36%)	–	–	–	–
Hospitalization	519 (0.16%)	1758 (0.54%)	2085 (0.04%)	5589 (0.10%)	–	–	–	–
Deaths	137 (0.04%)	334 (0.10%)	343 (0.01%)	839 (0.02%)	137 (26.4%)	334 (19.0%)	343 (16.5%)	839 (15.0%)
Admission to ICU	87 (0.03%)	217 (0.07%)	265 (0.00%)	554 (0.01%)	87 (16.8%)	217 (12.3%)	265 (12.7%)	554 (9.9%)

*Persons at risk was slightly different for this analysis as only the first positive test is present in the MIBA database. Positive tests before the start of the wave is therefore excluded. Persons with diabetes: wave 1 (n=321,933) and wave 2 (n=324,316). Persons without diabetes: wave 1 (n=5,479,752) and wave 2 (5,414,646) COVID-19 infection was defined by the first positive PCR test.

**Table 3:** Hazard ratio (HR) for persons with diabetes compared with persons without diabetes for COVID-19 related hospitalization, mortality, and admission to intensive care unit (ICU) in the whole Danish population, and COVID-19 related mortality and ICU admission in the hospitalized population only.

Outcome	HR (95% CI) crude		HR (95% CI) adjusted for age, sex		HR (95% CI) adjusted for age, sex and comorbidity		Interaction with wave, fully adjusted model
		Wave 1	Wave 2	Wave 1	Wave 2	Wave 1	Wave 2	
COVID-19 infection	1.53 (1.45, 1.62)	0.91 (0.89, 0.93)	1.58 (1.49, 1.67)	1.07 (1.05, 1.09)	1.36 (1.28, 1.45)	1.13 (1.11, 1.16)	p<0.001
Hospitalization	4.27 (3.87, 4.70)	5.29 (5.01, 5.58)	2.33 (2.11, 2.57)	2.90 (2.74, 3.07)	1.40 (1.27, 1.55)	1.76 (1.65, 1.87)	p<0.001
Mortality	6.84 (5.61, 8.34)	6.69 (5.89, 7.59)	2.39 (1.96, 2.92)	2.36 (2.07, 2.69)	1.65 (1.34, 2.03)	1.64 (1.43, 1.88)	p=0.95
Mortality in the hospitalized population	1.72 (1.41, 2.09)	1.29 (1.14, 1.47)	1.41 (1.16, 1.72)	1.19 (1.05, 1.35)	1.27 (1.03, 1.57)	1.07 (0.93, 1.23)	p=0.17
Admission to ICU unit	5.62 (4.41, 7.16)	6.58 (5.62, 7.70)	3.40 (2.64, 4.38)	3.99 (3.36, 4.75)	1.56 (1.20, 2.03)	1.85 (1.53, 2.23)	p=0.26
Admission to ICU in the hospitalized population	1.38 (1.09, 1.74)	1.26 (1.08, 1.47)	1.28 (1.01, 1.63)	1.18 (1.01, 1.39)	1.09 (0.84, 1.41)	1.02 (0.85, 1.21)	p=0.63

Mortality was defined as death within 30 days of the start of a COVID-19 related hospitalization, which was defined as hospitalization for more than 12 hours within 30 days prior and 14 days after a positive test for SARS CoV-2 and with a recorded COVID-19 diagnosis for the hospital contact.

**Figure 1:**
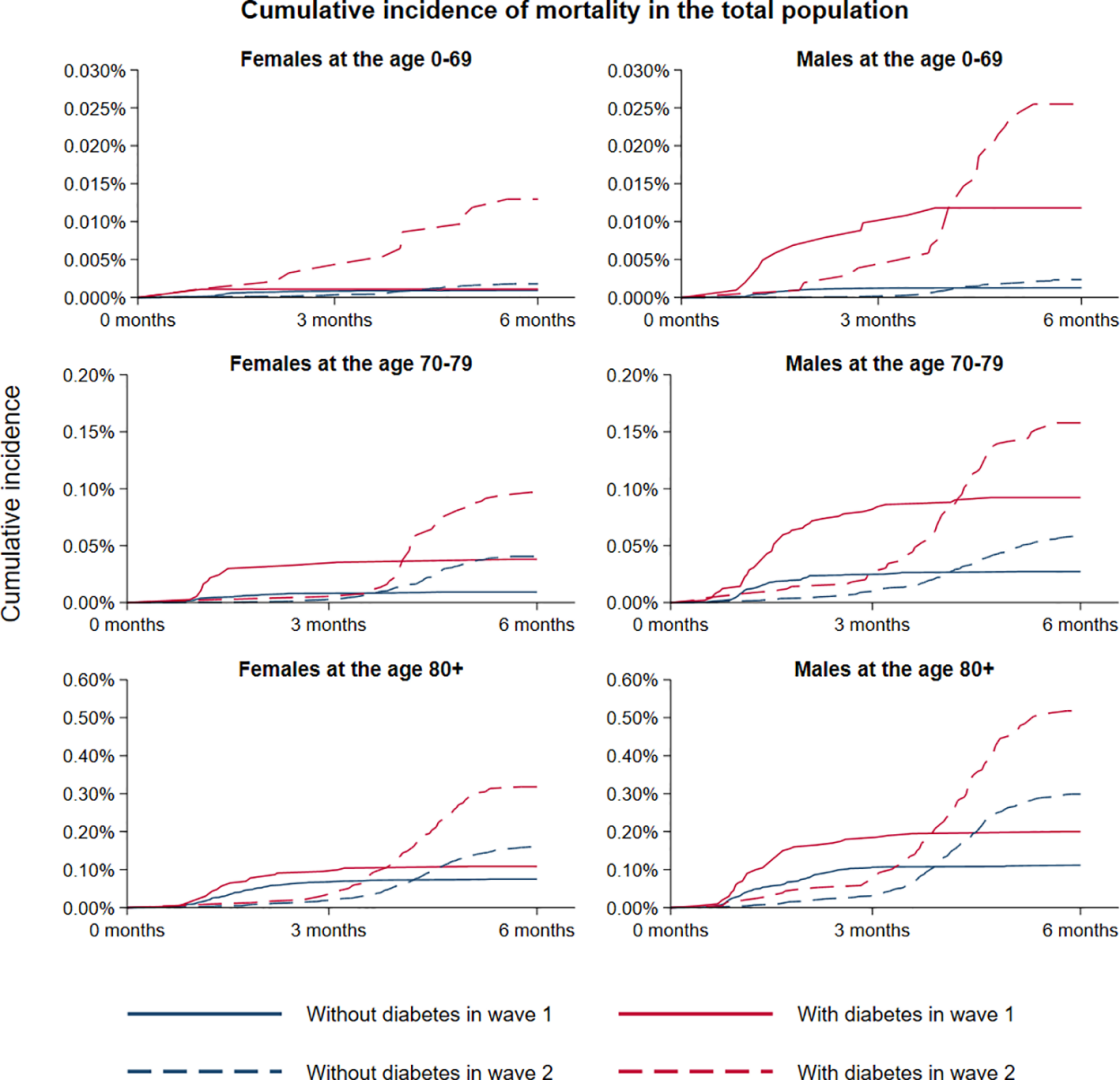
Crude mortality rates according to diabetes status during the first and second wave of COVID-19 in Denmark. Stratification were made according to females and males, and age 0-69, 70-79 and 80+ years, respectively. Time was defined according to the start date of the two waves.

We observed a total of 480 and 1173 deaths among persons hospitalized for COVID-19 in the first and second wave, respectively (**Table 2**). The crude 6-months mortality rate increased from 0.63 to 1.5 in 10,000 persons without diabetes and from 4.3 to 10 in 10,000 persons with diabetes from wave 1 to 2 (**Figure 1**, **ESM Figure 2**, **ESM Table 4**). HR for mortality, adjusted for sex and age, was 2.39 (95% CI 1.96, 2.92) and 2.36 (95% CI 2.07, 2.69) for persons with diabetes compared to persons without diabetes in the first and second wave, respectively (**Table 3**). In the fully adjusted model, the risk of death was also significantly increased in persons with diabetes compared to persons without diabetes, but the relative risk did not change between the two waves (HR 1.65; 95% CI 1.34, 2.03 and 1.64; 95% CI 1.43, 1.88; p=0.95 for interaction; **Table 3**).

When mortality was restricted to the hospitalized population only, the 6-months crude mortality rate decreased in persons with diabetes from 26.8% to 19.6% from the first to the second wave, while the corresponding mortality rate in persons without diabetes fell from 16.7% to 15.5% (**ESM Figure 3**, **ESM Table 4**). In the fully adjusted model, the mortality rate for persons with diabetes compared to persons without was increased in the first wave (HR 1.27; 95% CI 1.03, 1.57), while it was not increased in the second wave (HR 1.07; 95% CI 0.93, 1.23); p=0.17 for interaction with wave; **Table 3**).

As auxiliary analysis we estimated the rate of COVID-19 infection (**Table 2**). In wave 1 there was 16,892 COVID-19 cases, while there were 191,601 cases in wave 2. For persons with diabetes 1389 and 9896 had a COVID-19 infection in wave 1 and 2, respectively, corresponding to an infection rate of 0.43% and 3.05%. For persons without diabetes 15503 and 181705 persons experienced a COVID-19 infection, corresponding to an infection rate of 0.28% and 3.35% in wave 1 and 2, respectively. The risk of a COVID-19 infection was increased in persons with diabetes compared to persons without diabetes in both waves after adjustment for age and sex (HR 1.58; 95% CI 1.49, 1.67 and HR 1.07; 95% CI 1.05, 1.09). This was also seen in a fully adjusted model (HR 1.36; 95% CI 1.28, 1.45 and HR 1.13; 95% CI 1.11, 1.16). The risk was numerically higher in wave 1 compared to wave 2 (test for interaction not performed).

As a sensitivity analysis we also estimated the risk of a COVID-19 hospitalization given a positive COVID-19 PCR test (**ESM Table 5**). This validated our main analysis with an increased risk of being hospitalized in persons with diabetes in wave 1 and 2, in a fully adjusted model (HR 1.17; 95% CI 1.04, 1.32 and HR 1.57; 95% CI 1.47, 1.67). Mortality given a positive COVID-19 test was also increased in persons with diabetes in both wave 1 and 2 (HR 1.32; 95% CI 1.08, 1.60 and HR 1.28; 95% CI 1.15, 1.44), with no apparent difference between waves. The method used in the main analysis identified 480 deaths in wave 1 and 1173 in wave 2 compared to 624 and 1173 in the sensitivity analysis, corresponding to a difference of 144 and 582, respectively.

### Secondary outcome: Risk of ICU admission

Admission to ICU during a COVID-19 related hospitalization was seen in 352 and 771 persons in the first and second wave, respectively (**Table 2**). The crude 6-months cumulative incidence increased from 0.5 to 1.0 in 10,000 persons without diabetes and from 3 to 7 in 10,000 persons with diabetes from wave 1 to 2 (**ESM Figure 4**, **ESM Table 4**). The risk of admission to ICU was increased in persons with diabetes compared with persons without diabetes, and the relative risk numerically increased, from wave 1 to wave 2 (HR 1.56; 95% CI 1.20-2.03 and 1.85; 95% CI 1.53, 2.23, respectively; p=0.26 for interaction; **Table 3**). Restricting the analysis to the hospitalized population, the 6-months cumulative incidence of admission to ICU fell from 17.5% to 12.8% for persons with diabetes between the two waves, while it fell from 13.0% to 10.2% for persons without diabetes (**ESM Figure 5**, **ESM Table 4**). After full adjustment, the risk of ICU admission was not increased for persons with diabetes compared with persons without diabetes either in wave 1 or wave 2 when expressed according to the hospitalized population (HR 1.09; 95% CI 0.84, 1.41 and 1.02; 95% CI 0.85, 1.21; p=0.63 for interaction; **Table 3**)

### Stratified analyses

**Figure 2:**
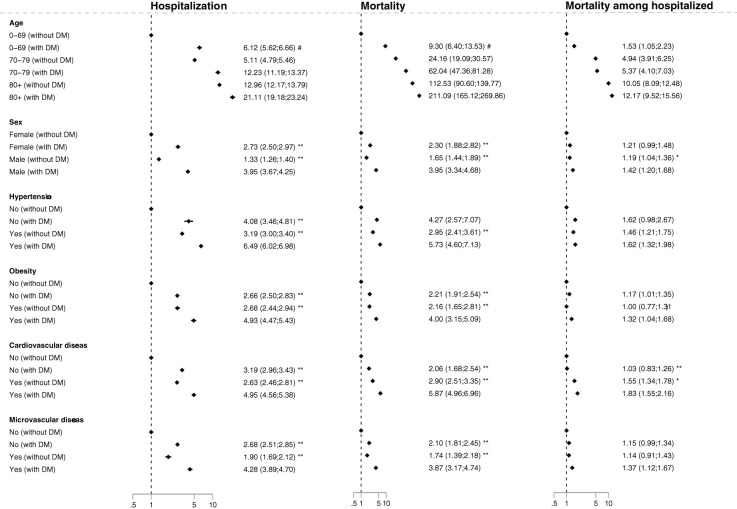
Forest plot of hazard ratios for the primary outcomes in persons with diabetes compared to persons without diabetes for stratified groups in wave 2, adjusted for age and sex. Mortality was defined as death within 30 days of the start of a COVID-19 related hospitalization, which was defined as hospitalization for more than 12 hours within 30 days prior and 14 days after a positive test for SARS-CoV-2 and with a recorded COVID-19 diagnosis for the hospital contact. Reference for the hazard ratio for the stratified groups were: 0-69 year without diabetes; females without diabetes; persons with neither hypertension nor diabetes; persons with neither obesity nor diabetes; persons with neither microvascular disease nor diabetes; persons with neither cardiovascular disease or diabetes, respectively. In addition the difference in HR was tested versus males with diabetes, hypertension with diabetes, obesity with diabetes, cardiovascular disease with diabetes, microvascular disease with diabetes, respectively: *p<0.05, **p<0.001. Difference in the risk between (the many) age groups and diabetes were tested overall by interaction analysis: # p<0.001.

All subgroup analyses presented here are from the second wave, whereas similar analyses for the first wave are given in **ESM Table 6** only. In the total population, age and diabetes had a strong interaction with respect to hospitalization and mortality (p<0001) with diabetes inferring the highest risk in the youngest age group (HR 6.12; 95% CI 5.62, 6.66 and 9.30; 95% CI 6.40, 13.53, respectively; **Figure 2** and **ESM Table 7**). However no significant overall interaction was present with respect to mortality in the hospitalized population, although younger persons with diabetes had an increased risk compared with persons without diabetes (HR 1.53; 95% CI 1.05, 2.23; **Figure 2** and **ESM Table 7**). In the total population, diabetes significantly increased the risk of hospitalization in both females (ref=1.0 vs HR 2.73; 95% CI 2.50, 2.97) and in males (HR 1.33; 95% CI 1.26, 1.40 and 3.95; 95% CI 3.34, 4.68, respectively; p<0.001 for difference between males with and without diabetes), with males overall having the highest risk (**Figure 2** and **ESM Table 7**). Diabetes also increased mortality risk both in females and males in the total population (**Figure 2**) as well as in the hospitalized population (females: ref=1.0 vs HR 1.21; 95% CI 0.99, 1.48 and males: HR 1.19; 95% CI 1.04, 1.36 vs HR 1.42; 95% CI 1.20, 1.68; p=0.04 for difference between males with and without diabetes).

Compared with persons without hypertension and without diabetes, persons with diabetes without hypertension had an increased risk of COVID-19 hospitalization (HR 4.08; 95% CI 3.46, 4.81), while persons with diabetes and hypertension had an even higher risk (HR 6.49; 95% CI 6.02, 6.96; p<0.001 versus diabetes without hypertension; **Figure 2** and **ESM Table 7**). Mortality was similarly increased in persons with diabetes without hypertension (HR 4.27; 95% CI 2.57, 7.07) and further increased in persons with diabetes with hypertension (HR 5.73; 95% CI 4.60, 7.13: P=0.26, **Figure 2** and **ESM Table 7**). However, when the mortality analysis was restricted to the hospitalized population, diabetes with hypertension did not increase the risk further compared to persons with diabetes without hypertension (HR 1.62; 95% CI 0.98, 2.67 vs HR 1.62; 95% CI 1.32, 1.98; p=0.99).

In the total population, diabetes without obesity conferred a higher risk of hospitalization compared to persons without diabetes and without obesity (HR 2.66; 95% CI 2.50, 2.83), while persons with both conditions had the highest risk (HR 4.93; 95% CI 4.47, 5.43; p<0.001 versus diabetes without obesity). The corresponding mortality was increased in persons with diabetes without obesity (HR 2.21; 95% CI 1.91, 2.54) and even higher in in persons with diabetes with obesity HR 4.00 (95% CI 3.15, 5.09; p<0.001 versus diabetes without obesity). Mortality in the hospitalized population was only increased in diabetes without obesity (HR 1.17; 95% CI 1.01, 1.35) and in persons with diabetes with obesity (HR 1.32; 95% CI 1.04, 1.68; p=0.11 versus diabetes without obesity).

In the total population, diabetes without cardiovascular disease also increased the risk of COVID-19 hospitalization (HR 3.19; 95% CI 2.96, 3.43) and mortality (HR 2.06; 95% CI 1.68, 2.54), while diabetes with cardiovascular disease increased the risk even further (HR 4.95; 95% CI 4.56, 5.38 and HR 5.87; 95% CI 4.96, 6.96, respectively, both p<0.001 versus diabetes without cardiovascular disease; **Figure 2** and **ESM Table 7**). However, in the hospitalized population, diabetes without cardiovascular disease did not increase mortality (HR 1.03; 95% CI 0.83, 1.26), whereas diabetes and cardiovascular disease still caused a higher mortality (HR 1.83; 95% CI 1.55, 2.16; p<0.001 versus diabetes without cardiovascular disease).

In the total population, diabetes without microvascular disease also increased the risk of COVID-19 hospitalization (HR 2.68; 95% CI 2.51, 2.85) and mortality (HR 2.10; 95% CI 1.81, 2.45), while diabetes with microvascular disease increased the risk even further (HR 4.28; 95% CI 3.89, 4.70 and HR 3.87; 95% CI 3.17, 4.74, respectively, both p<0.001 versus diabetes without microvascular disease; **Figure 2** and **ESM Table 7**). However, in the hospitalized population, diabetes without microvascular disease might not increase mortality (HR 1.15; 95% CI 0.99, 1.34), whereas diabetes and microvascular disease caused a higher mortality, although might not higher than in diabetes without microvascular disease (HR 1.37; 95% CI 1.12, 1.67; p=0.13 versus diabetes without cardiovascular disease).

The risk of ICU in the presented subgroups did not differ from the main results (**ESM Tables 8, 9**, **ESM Figure 4**). However, when restricted to the hospitalized population patients with more co-morbidity or high age had the lowest risk of being submitted at the ICU (**ESM Tables 8, 9**, **ESM Figure 5**).

The risk of hospitalization and the risk of ICU admission were higher in patients with type 2 diabetes compared with patients with type 1 diabetes after adjustment for age and sex (**ESM Table 10**). However, the mortality did not differ among persons with type 1 and type 2 diabetes after adjustment for sex and age.

## Discussion

As reported previously, diabetes increased the risk of COVID-19 related hospitalization and death compared to persons without diabetes. As a novel finding, we here report that the risk of COVID-19 hospitalization increased more in patients with diabetes than persons without diabetes from the first to the second wave, whereas the relative risk of mortality did not change due to a relative decrease in mortality among hospitalized patients with diabetes. Although the relative mortality risk did not change between the waves, the absolute increase in the cumulative incidence was much higher in persons with diabetes. Comparing individuals without hypertension, obesity, cardiovascular disease or microvascular disease, diabetes per se still conferred a higher risk of hospitalization and mortality, and these risks were further enhanced by the presence of each of these conditions. However, specifically, the risk of mortality in the hospitalized population was not increased in persons with diabetes without cardiovascular disease, while persons with both diabetes and cardiovascular disease had a particularly high mortality.

The ability to protect the population from severe COVID-19 infection and death is a primary concern in a pandemic. The risk of COVID-19 related death after hospitalization is determined by two components: 1) the risk of severe COVID-19 infection leading to hospitalization in the total population and 2) the risk of death among those hospitalized, as a marker of the effectiveness of the treatment of severe COVID-19. Despite the relative risk of COVID-19 related death was the same in wave 1 and 2 for persons with diabetes, this component analysis determined that the relative risk of hospitalization was further increased in wave 2. The absolute risk also increased substantially more in persons with diabetes compared to those without diabetes from wave 1 to wave 2. This suggests that the societal measures to prevent COVID-19 infection and hospitalization in susceptible individuals were more effective during wave 1. The specific reasons for this change cannot be completely ascertained. However, overall we consider the difference in societal measures to be most important for the reported change. During wave 1 a general and early lock-down on 11 March 2020, was effectuated in Denmark, including closing of schools, work at home for private and public employees in non-essential functions and a general minimization of societal activity (24). During wave 2 societal measures were more modest and selective and a national lock-down were enforced later in the COVID-19 second surge on 16 December 2020 (25, 26). The lockdown of the first wave were implemented on 11 March 2020 when the incidence of COVID-19 infections had risen from 10 per day on 7 March to 250 per day on 10 March and correspondingly the number of person in hospital due to COVID-19 were 19 on the 12 March (27). This was in contrast to the second wave were incident COVID-19 infection were 3692 per day on 16 December (28), while 404 were in hospital with COVID-19 and 57 were in the ICU (29). Other differences were that childcare services of preschool children were not included in the lockdown of wave 2 and that contact tracing and free access to testing were a part of the measures all through wave 2, a measure that was only part of wave 1 in later stages (30). An overview of the characteristics of the two waves are presented in **ESM Table 11**.

We have not identified any major changes in infectivity or virulence in the literature that are specific for persons with diabetes from wave 1 to wave 2, although a minor effect of the alpha variant cannot be ruled out as discussed below. We did not identify any changes in knowledge about the risk of people with diabetes, or in management of persons with diabetes within the first year of the pandemic. General information to high-risk groups including persons with diabetes and their treating physicians about the increased risk of severe COVID-19 infection were promoted by the national Danish health authorities, also very early in the pandemic at 12 March 2020 (31). During the two first waves it consisted of intensification of the general advice of hand disinfection, limited physical contact, distance during social activities and cleaning, coupled with advice for relatives to persons at high risk (https://www.sst.dk/en/English/Corona-eng/Prevent-infection/People-at-higher-risk). Despite these measures, the risk of COVID-19 related hospitalization rose more in persons with than without diabetes, which indicate that this high risk population benefited more from an early and general lock-down and that selective advice for this group did not alleviate an increase in risk seen during less societal restriction.

We have showed that both the risk of COVID-19 infection and the risk of a severe COVID-19 infection leading to hospitalization, given a positive PCR test, was increased in persons with diabetes (13 and 57% excess, respectively in wave 2). This indicate that persons with diabetes have a higher susceptibility to COVID-19 infection and in addition more susceptible to develop severe complications once infected. The reported decrease in risk of COVID-19 infection in persons with diabetes from wave 1 to wave 2 must considered to be highly biased, due to the very high difference in test activity and any firm conclusions on the change over time cannot be given.

Our results confirm the increased risk of mortality among hospitalized patients with diabetes (32), but the relative decrease of this apparent risk in hospitalized patients with diabetes compared with persons without diabetes from one to a subsequent COVID-19 wave is new. This adds to prior reports of a decrease in the mortality among hospitalized persons between COVID-19 waves (33, 34), but our results indicate that this is especially pronounced in diabetes. The increased use of corticosteroids (35), prone positioning (36) and anti-viral treatment (37) during the pandemic could have had an impact on the risk of mortality in hospitalized persons. As the risk of severe COVID-19 infection is higher in persons with diabetes, a higher impact of advances in the treatment of severe COVID-19 would concomitantly be expected in persons with diabetes. On the other hand, an increased alertness towards increased susceptibility in diabetes patients perhaps increased the chance of being hospitalized with milder COVID-19 infection in wave 2. However, our data do not suggest that more mild cases of COVID-19 in persons with diabetes were hospitalized in wave 2, as the absolute and relative risk of ICU admission in the total population were numerically higher in wave 2 compared to wave 1 in persons with diabetes, indicating a higher burden of severe COVID-19 infection in wave 2.

Long term COVID-19 symptoms (so-called long COVID) are present in some patients and are associated to severity of the initial COVID-19 infection (38–40), although inflammatory biomarkers do not seem to be increased 6 months after a COVID-19 infection (41). However, the association between long COVID and diabetes are not clear, with some studies reporting a significant association (42–44) and others not (38, 40, 45, 46), with most studies done in patients with a prior COVID-19 hospitalization. However, our data would suggest that the higher risk of hospitalization for persons with diabetes, would also translate into a higher risk of having long COVID, at least when evaluated in the total population. Therefore, it could be hypothesized that the diabetes population would have a higher burden of long COVID in general. Further studies are needed in well-defined non-selected cohorts, with appropriate adjustment for competing risk by death, to clarify this question.

We did not observe any difference in COVID-19 mortality between type 1 and type 2 diabetes, which is in accordance with other studies (32). We did observe a higher risk of hospitalization in type 2 diabetes. However, the number of hospitalizations and deaths among persons with type 1 diabetes constituted only 2.8% and 2.7% of the events in persons with diabetes and the results should therefore be interpreted cautious.

The strength of our study is the complete nationwide coverage of the comprehensive national registries and a very high degree of registration of results of RT-PCR testing for SARS-CoV-2 (47). Furthermore, we analyzed multiple aspects of the risk of severe COVID-19 outcomes, thereby elucidating the complexity of progress or regress in persons with diabetes.

The study has some limitations. During the second wave the lineage B.1.1.7 emerged (the alpha strain), and gradually became more prevalent. In week 53 of 2020 only 2% of sequenced cases were B.1.1.7, while the prevalence was 81.3% in week 9 of 2021 (https://www.covid19genomics.dk/statistics). The B.1.1.7 lineage was associated with an increased risk of transmission (48), increased risk of hospitalization (25) and increased risk of mortality (49, 50), which could have had an impact on the differences seen between wave 1 and 2. The two waves were defined in Spring and Summer (wave 1) and Autumn and Winter (wave 2), which could also affect the risk of transmission (51, 52), however we do not expect that this would affect persons with and without diabetes differently. During the first year of the pandemics the PCR test capacity in Denmark gradually increased from 2000/day on 21.april 2020 to 170.000/day on 2. March 2021 (https://tcdk.ssi.dk/om-testcenter-danmark/testkapacitet-gennem-tiden). We did not express the risk according to SARS-CoV-2 RT-PCR positivity due to the large difference in test activity, which would have had a large impact on the risk estimates. We would suspect that a much more restrictive test strategy would favor testing of high risk persons with eg diabetes, which we would expect to falsely increase the risk of a positive test in persons with diabetes compared to persons without diabetes. This bias will be lower for the hospitalized population, as their admission most considered based on more objective criteria. Therefore, we chose to define the population according to those hospitalized with COVID-19, as this population was expected to have close to complete coverage of screening for COVID-19. This strategy would possibly miss deaths from COVID-19 in persons not hospitalized or not tested for COVID-19, e.g. in persons in nursing homes. However, the proportion of persons which by clinical judgement are chosen not to be hospitalized, is expected to be rather constant. To assess the impact of these considerations, we also assessed the risk of hospitalization and death according to the test positive population. This sensitivity analysis confirmed the main results, indicating that these limitations did not impact the results. To further overcome these limitations, we both expressed the risk according to the whole population and according to the COVID-19 hospitalized population. As both mortality in the total and hospitalized population had a COVID-19 hospitalization as a prerequisite the two ways of assessing data are comparable. It is also important to recognize that at times of very high infection rates, many deaths, which would also had happened without COVID-19 being present (e.g accidents), by chance will happen after a positive COVID-19 test. When infection rates are very high, such deaths might constitute a high proportion of deaths within 30 days of a positive test, especially when the mortality of the infection at hand is relatively low. By restricting the analysis to persons being hospitalized with a COVID-19 diagnosis, this bias will be reduced.

Another limitation of the study is the lack of data on vaccination status which precludes assessing the impact of vaccination. Moreover, we did not assess the risk of long COVID-19, due to the difficult task of providing data of long COVID-19 from registries (53). Finally, the specific cause of mortality in COVID-19 related deaths in the hospitalized populations were unavailable.

In conclusion, we found that the risk of COVID-19 related hospitalization in the total Danish population increased more for persons with than without diabetes from wave 1 to 2 - but that the relative risk of mortality in hospitalized patients with diabetes actually decreased compared with persons without diabetes. Moreover, diabetes related co-morbidities as hypertension, obesity, microvascular disease and cardiovascular disease all further increased the risk of COVID-19 related hospitalization and death beyond diabetes. Any lessons learned from the first wave – about protective measures – did not reduce risk of hospitalization for persons with diabetes, but the reduced relative risk of mortality in hospitalized diabetes patients, shows that some improvement in treatment and care apparently equalized the differences. This implies that there is a need for better measures that specifically protect populations of high risk against COVID-19 hospitalization.

## Data availability statement

The datasets presented in this article are not readily available because sharing of participant data from public registries are not allowed by Danish law. Requests to access the datasets should be directed to kontakt@sundhedsdata.dk.

## Ethics statement

The study was approved by The Danish Data Protection Agency (Region of Southern Denmark, record no. 20/19971). Data were managed at The Danish Health Data Authority (project ID no. FSEID-00005015), who also approved the study and access to data. Ethical review and approval was not required for the study on human participants in accordance with the local legislation and institutional requirements. Written informed consent for participation was not required for this study in accordance with the national legislation and the institutional requirements.

## Author contributions

JS, AG and KH conceived and designed study. JS prepared the first draft. LR performed the statistical analysis. All authors contributed to the interpretation of data and critically revised the manuscript. JS, AG and KH are the guarantors of the work and accepts full responsibility for the work and/or the conduct of the study, had access to the data, and controlled the decision to publish. All authors contributed to the article and approved the submitted version.

## Funding

The study was funded by the Danish Agency for Higher Education and Science (Grant 0237-00012B). The study sponsor/funder was not involved in the design of the study; the collection, analysis, and interpretation of data; writing the report; and did not impose any restrictions regarding the publication of the report.

## Acknowledgments

We thank Inge Petersen, Open Patient data Explorative Network, Odense University Hospital for help with data management and statistical analyses.

## Conflict of interest

The authors declare that the research was conducted in the absence of any commercial or financial relationships that could be construed as a potential conflict of interest.

## Publisher’s note

All claims expressed in this article are solely those of the authors and do not necessarily represent those of their affiliated organizations, or those of the publisher, the editors and the reviewers. Any product that may be evaluated in this article, or claim that may be made by its manufacturer, is not guaranteed or endorsed by the publisher.

## Abbreviations

aHR, Adjusted hazard ratio; COVID-19, Coronavirus disease-2019; CRS, The Danish Civil Registration System; DNPR, Danish National Patient Registry; ICU, Intensive care unit; NPR, National Prescription Registry; MIBA, The Danish Microbiology Database; PCR, Polymerase Chain Replication; SARS-CoV-2, Acute respiratory syndrome coronavirus 2.
