# Supplementary material for: Risk of severe COVID-19 infection in persons with diabetes during the first and second waves in Denmark: A nationwide cohort study
Source: Front Endocrinol (Lausanne). 2022 Oct 11;13:1025699. doi: 10.3389/fendo.2022.1025699 (PMC9592709; doi:10.3389/fendo.2022.1025699)

Electronic supplementary material

Risk of severe COVID-19 infection in persons with diabetes during the first and second waves in Denmark: a nationwide cohort study

ESM Table 1. Definition of outcomes. Abbreviations: MIBA: The Danish Microbiology Database. DNPR: Danish National Patient Registry. NPR: National Prescription Registry. CRS: The Danish Civil Registration System

Outcome	Definition and codes	Sources
COVID-19 related hospitalization	Any positive COVID-19 test result (**Casedefinition =1**) AND any COVID-19 diagnosis (**DB342, DB972, DZ038PA1**) AND (hospitalization >=12 hours starting within 14 days after the positive test date (**SampleDate**) OR a hospitalization starting 30 days prior to **Sampledate**)<br>Date of the outcome is the ***first*** contact date (**STARTTIDSPUNKT**) that fulfills the above	**MIBA**<br><br>Variables: Casedefinition and SampleDate<br><br>**DNPR (version 3)**<br><br>*TABLE:KONTAKT*<br>Variables: STARTTIDSPUNKT (time stamp for first registration and start of contact). SLUTTIDSPUNKT (time stamp for end of contact)
COVID-19 related UCI contact	COVID-19 related hospitalization and admission in ICU within 30days of the index date. Admission to ICU was defined by procedure codes NABE	**DNPR (version 3)**<br><br>*TABLE: PROCEDURER*<br>Variables: KODE<br><br>*TABLE: PROCEDURER_TILLAEG*<br>Variables: TILLAEGSKODE
COVID-19 related death	COVID-19 related hospitalization and registration of death (**c_status=90** and **d_status_hen_start**= date of death) within 30 days of the index date	**CRS**<br><br>*Tabel: t_person*<br>Variables: c_status=90 and d_status_hen_start<br><br>**DNPR (version 3)**<br><br>*TABLE:KONTAKT*<br>Variables: STARTTIDSPUNKT (time stamp for first registration and start of contact). SLUTTIDSPUNKT (time stamp for end of contact).

ESM Table 2. Definition of exposure and co-morbidity groups. For first wave the exposure groups are defined by registrations until 1.3.2020 and until 1.9.2020 for the second wave.

Group	Definition and codes	Sources
Diabetes	One or more diabetes defining registration in DNPR including A and B-diagnoses and in- and outpatient contacts: ICD8: 249, 250 OR ICD10: DE10- DE14, DG632, DG590, DH280, DH334B, DH360, DM142, DN083, DO24 (except DO244, although it does not overrule other hits, see exceptions) OR One or more redemptions of glucose-lowering medication with ATC-codes: A10A, A10B<br>No time constraints were applied.<br><br>**Exceptions:**<br>1) A10BJ02 liraglutide with the tradename Saxenda cannot solely define the patient as having diabetes. Saxenda and victoza can be separated by the "varenummer"<br>2) A10BF cannot solely define the patient as having diabetes (acarbose)<br>3) Females below 50 years with diabetes defined exclusively by A10BA02 and that additionally have polycystic ovarie syndrome defined by ICD-10 codes DE282<br>4) ATC registrations of A10A in periods with pregnancy are not defining from date of birth (ICD-10 codes DO80-DO84) minus 30 weeks	**DNPR (version 2)**<br><br>*TABLE: t_diag*<br>Variables: c-diag (diagnosis codes) and c_diagtype (diagnosis type, =A or B)<br><br>*TABLE: t_adm*<br>Variables: c_pattype (contact type, =0, 1, 2 or 3) and d_inddto (time stamp for first registration)<br><br>**DNPR (version 3)**<br><br>*TABLE: DIAGNOSE*<br>Variables: KODE (diagnosis) and ART (diagnosis type =A or B)<br><br>*TABLE:KONTAKT*<br>Variables: STARTTIDSPUNKT (time stamp for first registration)<br><br>**NPR**<br><br>Variables: atc (atc-code), ovnr (varenummer) and etid (time of redemption)
Type 1 diabetes	All of the following A-C:<br>A) 2 or more redemptions of 10A except A10AE54 and A10AE56<br>B) No redemptions of A10B or (A10AE54 and A10AE56)<br>C) At least one registration of DE10 or 249 (ICD-8) (primary or secondary)	
Type 2 diabetes	All with diabetes not having type 1 diabetes	
Hypertension	One or more ICD-10 codes (including A and B-diagnoses and in- and outpatient contacts): DI10-DI15, DH350H, DI674 OR one or more redemptions of antihypertensive medication, ATC-codes: C02, C03A, C03B, C03D, C03E (except C03EB), C07, C08, C09A, C09B, C09C, C09D, C09X, G04CA03, C10BX03, C10BX04, C10BX06, C10BX07, C10BX09, C10BX10, C10BX11, C10BX12,	**DNPR (version 2)**<br><br>*TABLE: t_diag*<br>Variables: c-diag (diagnosis codes) and c_diagtype (diagnosis type, =A or B)<br><br>*TABLE: t_adm*<br>Variables: c_pattype (contact type, =0, 1, 2 or 3) and d_inddto (time stamp for first registration)

	C10BX13, C10BX14, C10BX15, C10BX16, C10BX17 and C10BX18<br>No time constraints were applied.	**DNPR (version 3)**<br><br>*TABLE: DIAGNOSE*<br>Variables: KODE (diagnosis) and ART (diagnosis type =A or B)<br><br>*TABLE:KONTAKT*<br>Variables: STARTTIDSPUNKT (time stamp for first registration)<br><br>**NPR**<br><br>Variables: atc (atc-code), ovnr (varenummer) and etid (time of redemption)
Obesity	One or more ICD-10 codes DE66 OR DZ980 OR operation codes for bariatric surgery: KJDF, KJAW90, KJAW91, KJDW96A, KJDW97A OR one or more redemptions of weight lowering medication with ATC code A10BJ02 (liraglutide with the tradename Saxenda; Saxenda and victoza can be separated by the "varenummer")	**DNPR (version 2)**<br><br>*TABLE: t_diag*<br>Variables: c-diag (diagnosis codes), c_diagtype (diagnosis type, =A or B) and c_tildiag<br><br>*TABLE: t_adm*<br>Variables: c_pattype (contact type, =0, 1, 2 or 3) and d_inddto (time stamp for first registration)<br><br>*TABLE: t_sksopr*<br>Variables: c_opr (operation codes) and c_tilopr (tillægs codes)<br><br>**DNPR (version 3)**<br><br>*TABLE: DIAGNOSE*<br>Variables: KODE (diagnosis) and ART (diagnosis type =A or B)<br><br>*TABLE:KONTAKT*<br>Variables: STARTTIDSPUNKT (time stamp for first registration)<br><br>*TABLE: DIAGNOSE_TILLAEG*<br>Variables: TILLAEGSKODE (addtional codes)<br><br>*TABLE: PROCEDURER*

		<u>Variables:</u> KODE (operation codes)<br><br>*TABLE: PROCEDURER_TILLAEG*<br><u>Variables:</u> TILLAEGSKODE (additional codes)<br><br><br>**NPR**<br><br><u>Variables:</u> atc (atc-code), ovnr (varenummer) and etid (time of redemption)
Cardiovascular disease	Ischemic heart disease (diagnosis codes: DI21, DI23, DI24, DI200 AND/OR Operation codes: KFNA, KFNB, KFNC, KFND, KFNE, KFNF, KFNG, KFLF)<br>Cerebrovascular disease (Diagnosis codes: DI60, DI61, DI63, DI64, DG45 AND/OR Operation codes: KAAL10, KAAL11)<br>Heart failure (Diagnosis codes: DI50, DI110, DI130, DI132)<br>Peripheral or central ischemic disease (Diagnosis codes: DI74, DN280, DK550, DH340-DH342 AND/OR<br>Operation codes:<br>A) KPAE, KPBE, KPCE, KPDE, KPEE, KPFE, KPAU74, KPCU74, KPDU74. KPEU74, KPFU74, KPGU74,<br>B) KPAF, KPBF, KPCF, KPDF, KPEF, KPFF,<br>C) KPAH, KPBH, KPCH, KPDH, KPEH, KPFH, KPGH20+21+22+23+30+31+40+99<br>D) KPAN, KPBN, KPCN, KPDN, KPEN, KPFN KPCU82, KPDU82, KPEU82, KPFU82<br>E) KPAP, KPBP, KPCP, KPDP, KPEP, KPFP, KPCU83, KPDU83, KPEU83, KPFU83, KPGU83<br>F) KPDW, KPEW, KPFW<br>Amputations (operation codes: KNEQ, KNFQ, KNGQ, KNHQ)	**DNPR (version 2)**<br><br>*TABLE: t_diag*<br><u>Variables:</u> c-diag (diagnosis codes), c_diagtype (diagnosis type, =A or B) and c_tildiag<br><br>*TABLE: t_adm*<br><u>Variables:</u> c_pattype (contact type, =0, 1, 2 or 3) and d_inddto (time stamp for first registration)<br><br>*TABLE: t_sksopr*<br><u>Variables:</u> c_opr (operation codes) and d_odto (time stamp for first registration)<br><br>**DNPR (version 3)**<br><br>*TABLE: DIAGNOSE*<br><u>Variables:</u> KODE (diagnosis) and ART (diagnosis type =A or B)<br><br>*TABLE:KONTAKT*<br><u>Variables:</u> STARTTIDSPUNKT (time stamp for first registration)<br><br>*TABLE: PROCEDURER*<br><u>Variables:</u> KODE (operation codes)<br><br>**NPR**<br><br><u>Variables:</u> atc (atc-code), ovnr (varenummer) and etid (time of redemption)

Microvascular disease	Chronic kidney disease (Diagnosis codes: DE18, DE102, DE112, DE132, DE142, DR809C, DR809D AND/OR Procedure codes: BJFD2 or BJFD0 for a period above 3 months AND minimum 2 registrations on average per week of or A-or B-diagnoses: DZ99.2) AND/OR Operation codes: KKAS00-20)<br><br>Diabetes related eye diseases (Diagnosis codes: DH360, DE103, DE113, DE123, DE133, DE143 OR Operation codes: KCKD05B, KCKC10, KCKC15)<br><br>Polyneuropathy (diagnosis codes; DG63.2, DG62.9 (A- and B-diagnoses)<br>DE10.4, DE11.4, DE12.4, DE13.4, -DE14.4 (only A-diagnoses))	**DNPR (version 2)**<br><br>*TABLE: t_diag*<br>Variables: c-diag (diagnosis codes), c_diagtype (diagnosis type, =A or B) and c_tildiag<br><br>*TABLE: t_adm*<br>Variables: c_pattype (contact type, =0, 1, 2 or 3) and d_inddto (time stamp for first registration)<br><br>*TABLE: t_sksopr*<br>Variables: c_opr (operation codes) and d_odto (time stamp for first registration)<br><br>**DNPR (version 3)**<br><br>*TABLE: DIAGNOSE*<br>Variables: KODE (diagnosis) and ART (diagnosis type =A or B)<br><br>*TABLE:KONTAKT*<br>Variables: STARTTIDSPUNKT (time stamp for first registration)<br><br>*TABLE: PROCEDURER*<br>Variables: KODE (operation codes)<br><br>**NPR**<br><br>Variables: atc (atc-code), ovnr (varenummer) and etid (time of redemption)

ESM Table 3: Charlson categories modified (A- and B-diagnosis codes in the last 5 years until 1.3.2020 for the first wave and until 1.9.2020 for the second wave.)

Charlson index categories	Definition and codes (DNPR)
Dementia	DF00; DF01; DF02; DF03; DF051; DG30; DG311
Chronic pulmonary disease	DI278; DI279; DJ40; DJ41; DJ42; DJ43; DJ44; DJ45; DJ46; DJ47; DJ60; DJ61; DJ62; DJ63; DJ64; DJ65; DJ66; DJ67; DJ684; DJ701; DJ703
Connective tissue disease	DM05; DM06; DM315; DM32; DM33; DM34; DM351; DM353; DM360
Ulcer disease	DK25; DK26; DK27; DK28
Any liver disease	DB18; DK700; DK701; DK702; DK703; DK709; DK713; DK714; DK715; DK717; DK73; DK74; DK760; DK762; DK763; DK764; DK768; DK769; DZ944; DI850; DI859; DI864; DI982; DK704; DK711; DK721; DK729; DK765; DK766; DK767
Hemiplegia	DG041; DG114; DG801; DG802; DG81; DG82;<br>DG830; DG831; DG832; DG833; DG834; DG839
Any cancer	DC00 - DC26; DC30 - DC34; DC37 - DC41;<br>DC43; DC45 - DC58; DC60 - DC76; DC77; DC78; DC79; DC80; DC81 - DC85; DC88; DC90 - DC97

ESM Table 4. Crude 6 month cumulative incidences (95% CI) during the first and second wave of COVID-19 in Denmark: 1) COVID-19 hospitalization, 2) death within 30 days of COVID 19 hospitalization (total population), 3) death within 30 days of COVID-19 hospitalization (hospitalized population), 4) intensive care unit (ICU) admissions within 30 days of hospitalization (total population), 5) ICU admissions within 30 days of hospitalization (hospitalized population), stratified according to diabetes status.

	6 month crude cumulative incidence, Wave 1 (%)	6 month crude cumulative incidence, wave 2 (%)
**COVID-19 hospitalization**		
Without diabetes	0.038 (0.036, 0.040)	0.10 (0.10, 0.11)
With diabetes	0.16 (0.15, 0.18)	0.54 (0.52, 0.57)
**Death within 30 days of COVID 19 hospitalization (total population)**		
Without diabetes	0.0063 (0.0056, 0.0070)	0.015 (0.014, 0.017)
With diabetes	0.043 (0.036, 0.050)	0.10 (0.09, 0.11)
**Death within 30 days of COVID19 hospitalization (hospitalized population)**		
Without diabetes	16.66 (15.08, 18.30)	15.46 (14.51, 16.44)
With diabetes	26.82 (23.05, 30.73)	19.64 (17.79, 21.57)
**ICU admissions within 30 days of hospitalization (total population)**		
Without diabetes	0.0048 (0.0043, 0.0055)	0.010 (0.009, 0.011)
With diabetes	0.027 (0.022, 0.033)	0.067 (0.058, 0.076)
**ICU admissions within 30 days of hospitalization (hospitalized population)**		
Without diabetes	13.03 (11.6, 14.5)	10.22 (9.43, 11.05)
With diabetes	17.45 (14.2, 20.9)	12.79 (11.2, 14.4)

ESM Table 5: Hazard ratio (HR) for persons with diabetes compared with persons without diabetes for COVID-19 related hospitalization and mortality, in the COVID-19 infected population only. Mortality was defined as death within 30 days of the start of a COVID-19 related hospitalization, which was defined as hospitalization for more than 12 hours within 30 days prior and 14 days after a positive test for SARS CoV-2 and with a recorded COVID-19 diagnosis for the hospital contact.

Outcome	HR (95% CI) crude		HR (95% CI) adjusted for age, sex		HR (95% CI) adjusted for age, sex and co-morbidity	
	Wave 1	Wave 2	Wave 1	Wave 2	Wave 1	Wave 2
Hospitalization	2.86 (2.57, 3.18)	6.14 (5.79, 6.50)	1.51 (1.35, 1.69)	2.49 (2.34, 2.65)	1.17 (1.04, 1.32)	1.57 (1.47, 1.67)
Mortality	4.35 (3.65, 5.19)	6.59 (5.92, 7.33)	1.52 (1.27, 1.82)	1.63 (1.46, 1.82)	1.32 (1.08;1.60)	1.28 (1.15;1.44)

ESM Table 6: Hazard ratios for the primary outcomes in persons with diabetes compared to persons without for specified subgroups in wave 1 adjusted for age and sex. Mortality was defined as death within 30 days of the start of a COVID-19 related hospitalization, which was defined as hospitalization for more than 12 hours within 30 days prior and 14 days after a positive test for SARS-CoV-2 and with a recorded COVID-19 diagnosis for the hospital contact.

	Total population	No of hospitalizations	No of deaths	Hospitalization, HR	Mortality, HR	Mortality (hospitalized pop) HR
Overall	5,801,688	2,604	480	2.42 (2.19, 2.68)	2.40 (1.96, 2.94)	1.40 (1.14, 1.71)
Age 0-69, non-diabetes	4,768,399	1,138	53	Reference*	Reference*	Reference
Age 0-69, diabetes	193,253	179	13	3.86 (3.30, 4.52)	5.98 (3.26, 10.97)	1.52 (0.83, 2.79)
Age 70-79, non-diabetes	480,363	426	84	3.77 (3.38, 4.22)	16.22 (11.50, 22.88)	4.54 (3.22, 6.41)
Age 70-79, diabetes	85,587	202	59	9.76 (8.40, 11.33)	60.02 (41.41, 86.99)	6.99 (4.82, 10.12)
Age 80+, non-diabetes	230,993	521	206	9.89 (8.92, 10.98)	87.46 (64.63, 118.37)	11.00 (8.12, 14.90)
Age 80+, diabetes	43,093	138	65	13.79 (11.55, 16.45)	141.64 (98.54, 203.59)	14.18 (9.86, 20.38)
Female, non-diabetes	2,765,179	973	152	Reference	Reference	Reference
Female, diabetes	151,559	199	40	2.22 (1.90, 2.59)*	1.90 (1.34, 2.69)*	1.11 (0.78, 1.58)*
Male, non-diabetes	2,714,576	1,112	191	1.30 (1.19, 1.41)*	1.69 (1.37, 2.09)*	1.37 (1.11, 1.70)*
Male, diabetes	170,374	320	97	3.34 (2.94, 3.80)	4.61 (3.57, 5.96)	2.17 (1.68, 2.81)
No hypertension, non-diabetes	4,183,775	892	68	Reference	Reference	Reference
No hypertension, diabetes	66,870	56	6	3.54 (2.70, 4.64)	3.34 (1.45, 7.73)	0.96 (0.42, 2.22)
Hypertension, non-diabetes	1,295,980	1,193	275	2.55 (2.30, 2.82)*	2.71 (2.00, 3.68)*	1.37 (1.03, 1.83)*
Hypertension, diabetes	255,063	463	131	4.50 (3.96, 5.10)	5.43 (3.90, 7.56)	1.88 (1.38, 2.56)
No obesity, non-diabetes	5,284,166	1,920	319	Reference	Reference	Reference
No obesity, diabetes	258,443	389	111	2.25 (2.01, 2.52)*	2.32 (1.86, 2.89)**	1.42 (1.14, 1.77)
Obesity, non-diabetes	195,589	165	24	2.46 (2.10, 2.89)*	2.23 (1.47, 3.38)	0.92 (0.60, 1.39)
Obesity, diabetes	63,490	130	26	4.04 (3.38, 4.84)	3.60 (2.41, 5.38)	1.26 (0.84, 1.89)
No cardiovascular disease, non-diabetes	5,172,221	1,614	200	Reference	Reference	Reference
No cardiovascular disease, diabetes	239,915	283	55	2.49 (2.18, 2.83)*	2.27 (1.67, 3.07)*	1.32 (0.97, 1.78)

Cardiovascular disease, non-diabetes	307,534	471	143	2.22 (1.98, 2.48)*	2.38 (1.90, 3.00)*	1.38 (1.10, 1.72)**
Cardiovascular disease, diabetes	82,018	236	82	4.00 (3.46, 4.63)	4.86 (3.71, 6.36)	1.84 (1.41, 2.39)
No microvascular disease, non-diabetes	5,412,910	1,983	302	Reference	Reference	Reference
No microvascular disease, diabetes	267,121	349	87	2.10 (1.87, 2.36)*	2.10 (1.65, 2.67)*	1.47 (1.15, 1.87)
Microvascular disease, non-diabetes	66,845	102	41	1.68 (1.37, 2.06)*	2.23 (1.60, 3.11)*	1.54 (1.10, 2.14)
Microvascular disease, diabetes	54,812	170	50	4.18 (3.56, 4.91)	4.45 (3.29, 6.03)	1.44 (1.07, 1.95)

*p<0.001, **p<0.05 against the last of the four subgroups. For age the p-value were depicting the propability of no interaction between diabetes and age.

ESM Table 7: Hazard ratios for the primary outcomes in persons with diabetes compared to persons without for specified subgroups in wave 2 adjusted for age and sex. Mortality was defined as death within 30 days of the start of a COVID-19 related hospitalization, which was defined as hospitalization for more than 12 hours within 30 days prior and 14 days after a positive test for SARS-CoV-2 and with a recorded COVID-19 diagnosis for the hospital contact.

	Total population	No of hospitalizations	No of deaths	Hospitalization, HR	Mortality, HR	Mortality (hospitalized pop) HR
Overall	5,755,057	7,347	1,173	2.86 (2.71, 3.03)	2.36 (2.08, 2.68)	1.20 (1.06, 1.36)
Age 0-69, non-diabetes	4,708,571	2,632	98	Reference*	Reference*	Reference
Age 0-69, diabetes	194,719	668	38	6.12 (5.62, 6.66)	9.30 (6.40, 13.53)	1.53 (1.05, 2.23)
Age 70-79, non-diabetes	485,259	1,359	237	5.04 (4.72, 5.38)	24.16 (19.09, 30.57)	4.94 (3.91, 6.25)
Age 70-79, diabetes	86,592	596	114	12.23 (11.19, 13.37)	62.04 (47.36, 81.28)	5.37 (4.10, 7.03)
Age 80+, non-diabetes	235,672	1,598	504	12.48 (11.73, 13.28)	112.53 (90.60, 139.77)	10.05 (8.09, 12.48)
Age 80+, diabetes	44,244	494	182	21.11 (19.18, 23.24)	211.09 (165.12, 269.86)	12.17 (9.52, 15.56)
Female, non-diabetes	2,740,780	2,593	379	Reference	Reference	Reference
Female, diabetes	153,327	691	123	2.73 (2.50, 2.97)*	2.30 (1.88, 2.82)*	1.21 (0.99, 1.48)
Male, non-diabetes	2,688,722	2,996	460	1.33 (1.26, 1.40)*	1.65 (1.44, 1.89)*	1.19 (1.04, 1.36)**
Male, diabetes	172,228	1,067	211	3.95 (3.67, 4.25)	3.95 (3.34, 4.68)	1.42 (1.20, 1.68)
No hypertension, non-diabetes	4,131,070	2,018	145	Reference	Reference	Reference
No hypertension, diabetes	67,802	151	17	4.08 (3.46, 4.81)*	4.27 (2.57, 7.07)	1.62 (0.98, 2.67)
Hypertension, non-diabetes	1,298,432	3,571	694	3.19 (3.00, 3.40)*	2.95 (2.41, 3.61)*	1.46 (1.21, 1.75)
Hypertension, diabetes	257,753	1,607	317	6.49 (6.02, 6.98)	5.73 (4.60, 7.13)	1.62 (1.32, 1.98)
No obesity, non-diabetes	5,228,339	5,100	780	Reference	Reference	Reference
No obesity, diabetes	260,744	1,311	261	2.66 (2.50, 2.83)*	2.21 (1.91, 2.54)*	1.17 (1.01, 1.35)
Obesity, non-diabetes	201,163	489	59	2.68 (2.44, 2.94)*	2.16 (1.65, 2.81)*	1.00 (0.77, 1.31)
Obesity, diabetes	64,811	447	73	4.93 (4.47, 5.43)	4.00 (3.15, 5.09)	1.32 (1.04, 1.68)
No cardiovascular disease, non-diabetes	5,119,696	4,075	448	Reference	Reference	Reference
No cardiovascular disease, diabetes	242,582	968	114	3.19 (2.96, 3.43)*	2.06 (1.68, 2.54)*	1.03 (0.83, 1.26)*

Cardiovascular disease, non-diabetes	309,806	1,514	391	2.63 (2.46, 2.81)*	2.90 (2.51, 3.35)*	1.55 (1.34, 1.78)**
Cardiovascular disease, diabetes	82,973	790	220	4.95 (4.56, 5.38)	5.87 (4.96, 6.96)	1.83 (1.55, 2.16)
No microvascular disease, non-diabetes	5,361,160	5,250	754	Reference	Reference	Reference
No microvascular disease, diabetes	270,084	1,261	223	2.68 (2.51, 2.85)*	2.10 (1.81, 2.45)*	1.15 (0.99, 1.34)
Microvascular disease, non-diabetes	68,342	339	85	1.90 (1.69, 2.12)*	1.74 (1.39, 2.18)*	1.14 (0.91, 1.43)
Microvascular disease, diabetes	55,471	497	111	4.28 (3.89, 4.70)	3.87 (3.17, 4.74)	1.37 (1.12, 1.67)

*p<0.001, **p<0.05 against the last of the four subgroups. For age the p-value were depicting the propability of no interaction between diabetes and age.

ESM Table 8: Hazard ratios for admission to intensive care unit (ICU) in persons with diabetes compared to persons without diabetes in the total and hospitalized populations for specified subgroups in wave 2. Admission to ICU was defined as ICU admission within 30 days of the start of a COVID-19 related hospitalization, which was defined as hospitalization for more than 12 hours within 30 days prior and 14 days after a positive test for SARS-CoV-2 and with a recorded COVID-19 diagnosis for the hospital contact.

	Total	No of ICU admissions	ICU (total population) Age- and sex-adjusted HR	ICU (hospitalized population) Age- and sex-adjusted HR
Overall	5,755,057	771	4.09 (3.46, 4.83)	1.20 (1.03, 1.41)
Age 0-69, non-diabetes	4,708,571	311	Reference	Reference
Age 0-69, diabetes	194,719	104	8.00 (6.41, 9.99)	1.32 (1.06, 1.65)
Age 70-79, non-diabetes	485,259	192	6.22 (5.19, 7.44)	1.21 (1.01, 1.45)
Age 70-79, diabetes	86,592	91	15.43 (12.22, 19.50)	1.26 (1.00, 1.59)
Age 80+, non-diabetes	235,672	51	3.65 (2.72, 4.92)	0.28 (0.21, 0.38)
Age 80+, diabetes	44,244	22	8.06 (5.23, 12.42)	0.38 (0.25, 0.59)
Female, non-diabetes	2,740,780	189	Reference	Reference
Female, diabetes	153,327	71	4.60 (3.49, 6.07)	1.42 (1.08, 1.87)
Male, non-diabetes	2,688,722	365	2.08 (1.75, 2.48)	1.61 (1.35, 1.92)
Male, diabetes	172,228	146	8.04 (6.44, 10.04)	1.79 (1.44, 2.22)
No hypertension, non-diabetes	4,131,070	193	Reference	Reference
No hypertension, diabetes	67,802	16	4.85 (2.91, 8.08)	1.09 (0.65, 1.81)
Hypertension, non-diabetes	1,298,432	361	4.88 (4.03, 5.90)	1.37 (1.14, 1.65)
Hypertension, diabetes	257,753	201	11.82 (9.53, 14.66)	1.52 (1.24, 1.87)
No obesity, non-diabetes	5,228,339	477	Reference	Reference
No obesity, diabetes	260,744	156	3.94 (3.26, 4.77)	1.24 (1.04, 1.49)
Obesity, non-diabetes	201,163	77	4.96 (3.88, 6.32)	1.80 (1.41, 2.30)
Obesity, diabetes	64,811	61	7.85 (5.99, 10.29)	1.36 (1.04, 1.78)
No cardiovascular disease, non-diabetes	5,119,696	427	Reference	Reference
No cardiovascular disease, diabetes	242,582	124	4.45 (3.61, 5.47)	1.18 (0.97, 1.45)
Cardiovascular disease, non-diabetes	309,806	127	2.87 (2.31, 3.56)	0.93 (0.76, 1.14)
Cardiovascular disease, diabetes	82,973	93	7.14 (5.60, 9.10)	1.17 (0.93, 1.48)

No microvascular disease, non-diabetes	5,361,160	522	Reference	Reference
No microvascular disease, diabetes	270,084	157	3.89 (3.23, 4.69)	1.23 (1.03, 1.47)
Microvascular disease, non-diabetes	68,342	32	2.71 (1.88, 3.91)	1.19 (0.83, 1.71)
Microvascular disease, diabetes	55,471	60	6.30 (4.78, 8.30)	1.18 (0.90, 1.55)

ESM Table 9: Hazard ratios for admission to intensive care unit (ICU) in persons with diabetes compared to persons without diabetes in the total and hospitalized populations for specified subgroups in wave 1. Admission to ICU was defined as ICU admission within 30 days of the start of a COVID-19 related hospitalization, which was defined as hospitalization for more than 12 hours within 30 days prior and 14 days after a positive test for SARS-CoV-2 and with a recorded COVID-19 diagnosis for the hospital contact.

	Total	No of ICU admissions	ICU (total population) Age- and sex-adjusted HR	ICU (hospitalized population) Age- and sex-adjusted HR
Overall	5,801,688	352	3.23 (2.50, 4.17)	1.21 (0.95, 1.56)
Age 0-69, non-diabetes	4,768,399	144	Reference	Reference
Age 0-69, diabetes	193,253	39	6.57 (4.61, 9.36)	1.70 (1.19, 2.42)
Age 70-79, non-diabetes	480,363	90	6.47 (4.97, 8.43)	1.74 (1.33, 2.26)
Age 70-79, diabetes	85,587	39	14.35 (10.07, 20.44)	1.57 (1.10, 2.23)
Age 80+, non-diabetes	230,993	31	5.00 (3.39, 7.37)	0.52 (0.35, 0.76)
Age 80+, diabetes	43,093	9	7.27 (3.71, 14.25)	0.56 (0.29, 1.10)
Female, non-diabetes	2,765,179	82	Reference	Reference
Female, diabetes	151,559	21	2.99 (1.84, 4.86)	1.18 (0.73, 1.92)
Male, non-diabetes	2,714,576	183	2.43 (1.87, 3.16)	1.89 (1.45, 2.45)
Male, diabetes	170,374	66	8.07 (5.79, 11.24)	2.31 (1.66, 3.21)
No hypertension, non-diabetes	4,183,775	93	Reference	Reference
No hypertension, diabetes	66,870	11	6.96 (3.72, 13.02)	2.01 (1.08, 3.77)
Hypertension, non-diabetes	1,295,980	172	4.56 (3.45, 6.03)	1.72 (1.31, 2.26)
Hypertension, diabetes	255,063	76	8.49 (6.09, 11.84)	1.70 (1.23, 2.34)
No obesity, non-diabetes	5,284,166	241	Reference	Reference
No obesity, diabetes	258,443	65	3.05 (2.29, 4.06)	1.22 (0.92, 1.62)
Obesity, non-diabetes	195,589	24	3.26 (2.14, 4.98)	1.25 (0.82, 1.91)
Obesity, diabetes	63,490	22	5.54 (3.56, 8.62)	1.29 (0.83, 2.00)
No cardiovascular disease, non-diabetes	5,172,221	212	Reference	Reference
No cardiovascular disease, diabetes	239,915	58	3.86 (2.85, 5.23)	1.48 (1.10, 1.98)
Cardiovascular disease, non-diabetes	307,534	53	2.04 (1.47, 2.83)	0.90 (0.66, 1.23)

Cardiovascular disease, diabetes	82,018	29	3.71 (2.46, 5.60)	0.83 (0.56, 1.24)
No microvascular disease, non-diabetes	5,412,910	251	Reference	Reference
No microvascular disease, diabetes	267,121	58	2.81 (2.09, 3.79)	1.22 (0.91, 1.63)
Microvascular disease, non-diabetes	66,845	14	2.29 (1.32, 3.97)	1.36 (0.79, 2.35)
Microvascular disease, diabetes	54,812	29	5.80 (3.90, 8.62)	1.27 (0.86, 1.87)

ESM Table 10: Hazard ratio (HR) for persons with type 1 diabetes and type 2 diabetes compared with persons without diabetes for COVID-19 related hospitalization, mortality, and intensive care unit (ICU) admission in the whole Danish population, and in mortality and ICU admission in the hospitalized population. Mortality was defined as death within 30 days of the start of a COVID-19 related hospitalization, which was defined as hospitalization for more than 12 hours within 30 days prior and 14 days after a positive test for SARS-CoV-2 and with a recorded COVID-19 diagnosis for the hospital contact.

Outcome	HR crude	HR adjusted for age, sex	Adjusted model, Interaction with type
Hospitalization, type 1 diabetes, n=49	1.90 (1.43-2.51)	1.97 (1.49-2.61)	P=0.01
Hospitalization, type 2 diabetes, n=1758	5.57 (5.28-5.88)	2.94 (2.77-3.12)	
Mortality, type 1 diabetes, n=9	2.32 (1.20-4.48)	2.62 (1.36-5.05)	p=0.75
Mortality, type 2 diabetes, n=325	7.06 (6.21-8.02)	2.36 (2.06-2.69)	
Mortality in the hospitalized population, type 1 diabetes	1.23 (0.65-2.32)	1.93 (1.02-3.66)	p=0.14
Mortality in the hospitalized population, type 2 diabetes	1.30 (1.14-1.47)	1.18 (1.04-1.34)	
ICU admission, type 1 diabetes	1.17 (0.38-3.65)	1.12 (0.36-3.47)	p=0.02
ICU admission, type 2 diabetes	7.03 (6.01-8.23)	4.15 (3.48-4.96)	
ICU admission in the hospitalized population, type 1 diabetes	0.61 (0.20-1.88)	0.53 (0.17-1.66)	p=0.16
ICU admission in the hospitalized population, type 2 diabetes	1.28 (1.10-1.50)	1.21 (1.03-1.41)	

ESM Table 11: Main characteristics of the protective measures in the first and second waves. Characteristics of the first wave are mainly adapted from lyngse et al (24), while other information are from references 26-31.

Measure	Wave 1	Wave 2
**Testing**		
All suspected cases	All wave	All wave
Severe symptoms	All wave	All wave
Healthcare workers	All wave	All wave
Contacts of cases	Late in wave	All wave
Free access for all residents	Late in wave	All wave
**Contact tracing**		
Centralised	Very late in wave	All wave
Patient Advice	Very late in wave	All wave
Contact tracing app	Very late in wave	All wave
**Workplaces**		
Smaller shops closed	Early in phase of exponential increase in cases	Late in phase of exponential increase in cases
Shopping malls closed	Early in phase of exponential increase in cases	Late in phase of exponential increase in cases
Restaurants and cafes closed	Early in phase of exponential increase in cases	Late in phase of exponential increase in cases
Many public workplaces closed	Early in phase of exponential increase in cases	Late in phase of exponential increase in cases
Staying at home encouraged	Early in phase of exponential increase in cases	Late in phase of exponential increase in cases
**Education and childcare**		
Child care	Early in phase of exponential increase in cases	Not closed
Schools (up to Grade 5) closed	Early in phase of exponential increase in cases	Late in phase of exponential increase in cases
Grade 5 to senior high schools closed	Early in phase of exponential increase in cases	Late in phase of exponential increase in cases
Universities closed	Early in phase of exponential increase in cases	Late in phase of exponential increase in cases
**Society**		
Local measures in areas with high infection rates	No	Yes
Ban on public assembly	All wave	Midway in the wave
Borders closed	First part of the wave	No
Events (e.g. sports) closed or restricted	First part of the wave	In door activities banned late in phase of exponential increase in cases
Face masks required in public domains	No	Yes
**Specific advice for persons at risk, including persons with diabetes**	Hand disinfection, limited physical contact, distance during social activities and	Hand disinfection, limited physical contact, distance during social activities and

	cleaning, coupled with advice for relatives to persons at high risk	cleaning, coupled with advice for relatives to persons at high risk
**Management of diabetes control**	Primary by virtual contacts	Primary by virtual contacts
**COVID-19 treatment of patients with diabetes**	Similar to persons without diabetes	Similar to persons without diabetes

ESM Figure 1A. Crude cumulative incidence of COVID-19 related hospitalization during the first and second wave of COVID-19 in Denmark stratified according to diabetes status. Time was defined according to the start date of the two waves.

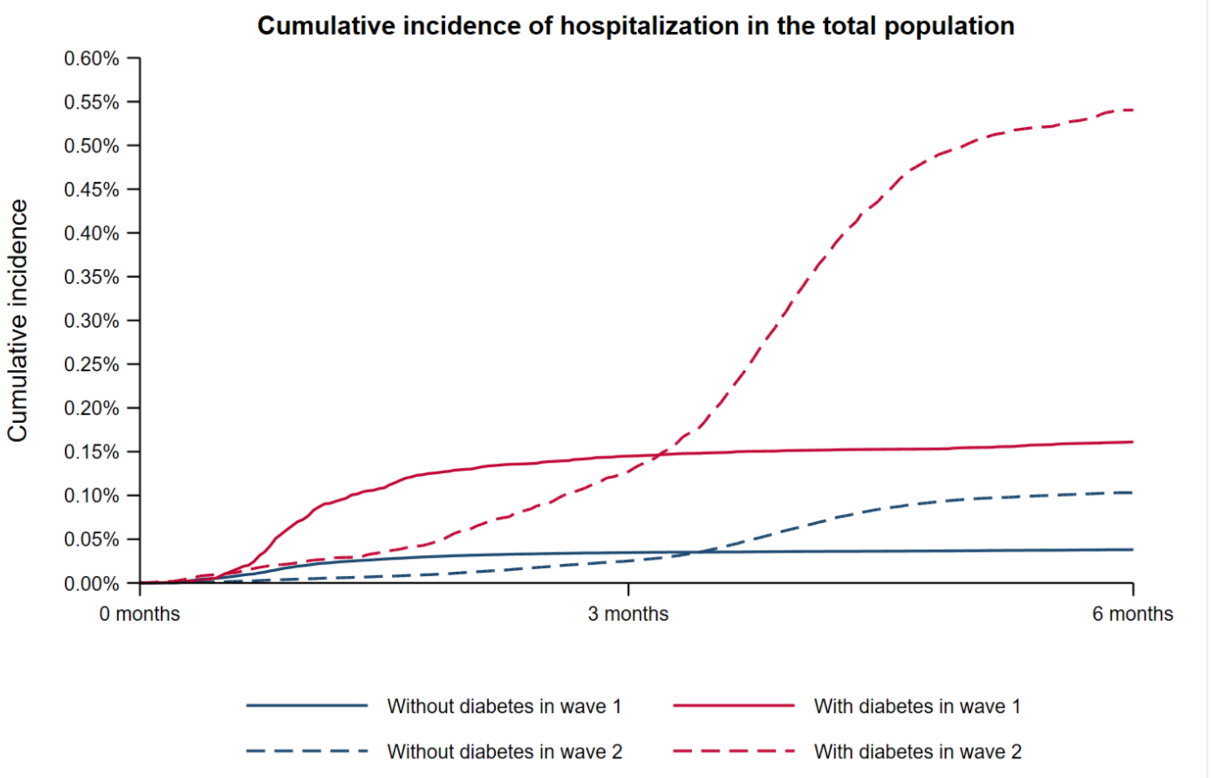

ESM Figure 1B. Cumulative incidence stratified by age and sex of COVID-19 related hospitalization during the first and second wave of COVID-19 in Denmark according to diabetes status. Time was defined according to the start date of the two waves.

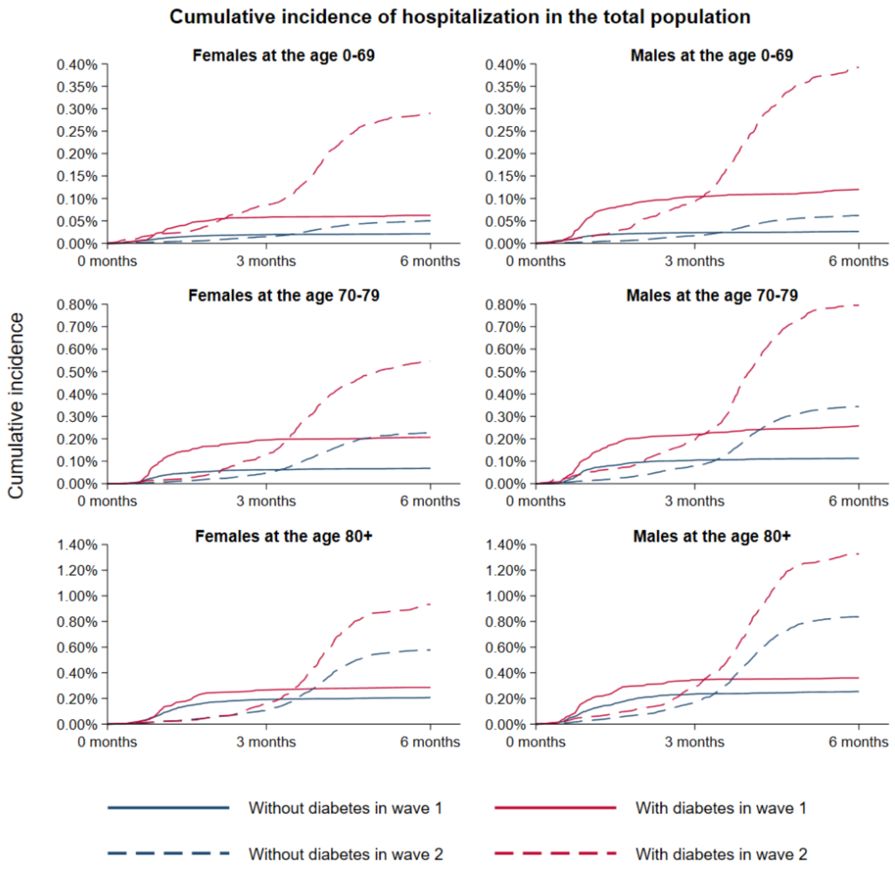

ESM Figure 2. Crude cumulative incidence of COVID-19 related mortality during the first and second wave of COVID-19 in Denmark stratified according to diabetes status.Time was defined according to the start date of the two waves.

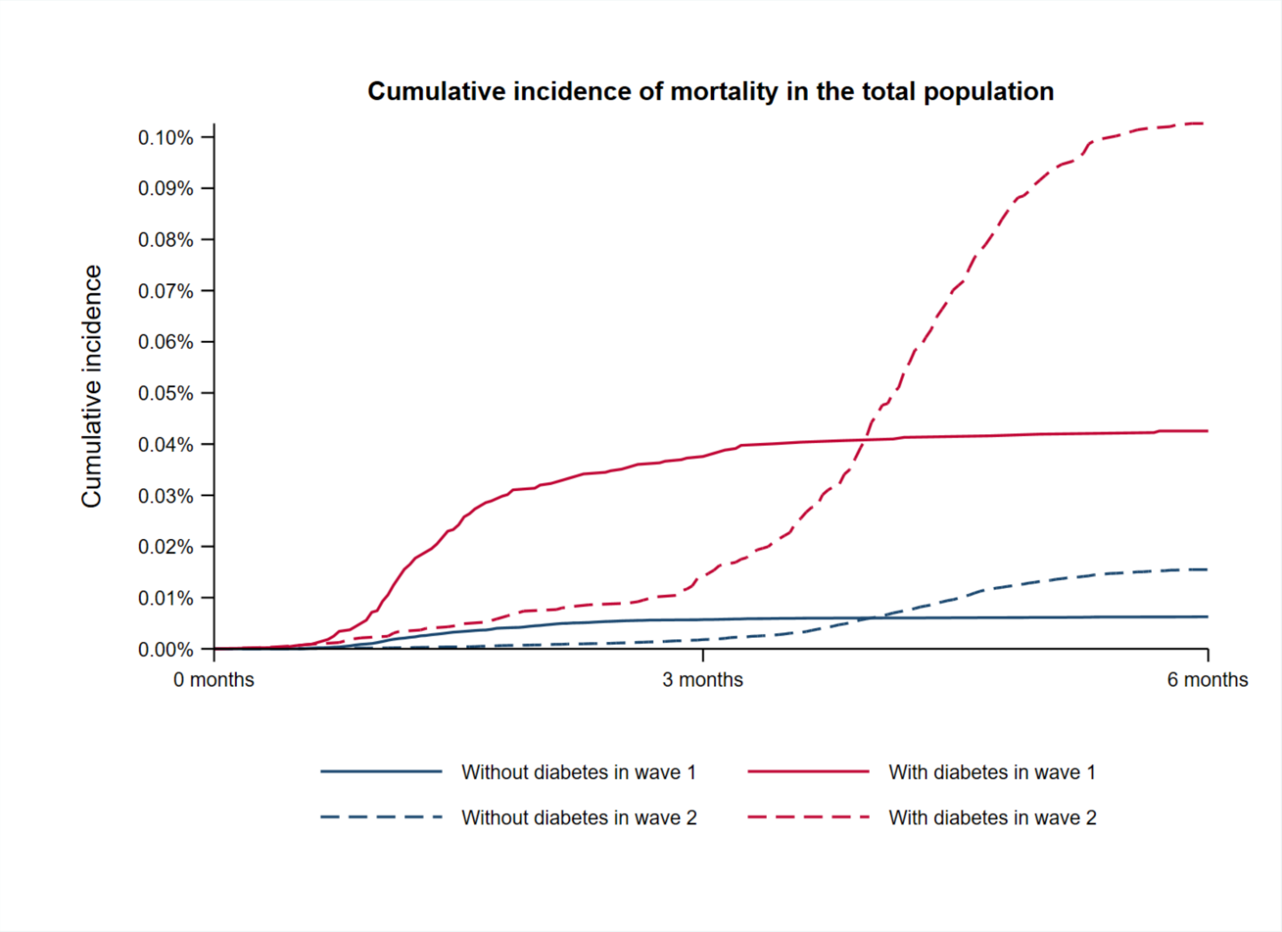

ESM Figure 3A. Crude COVID-19 related mortality in the hospitalized population during the first and second wave of COVID-19 in Denmark according to diabetes status. Time was defined according to the start date of hospitalization.

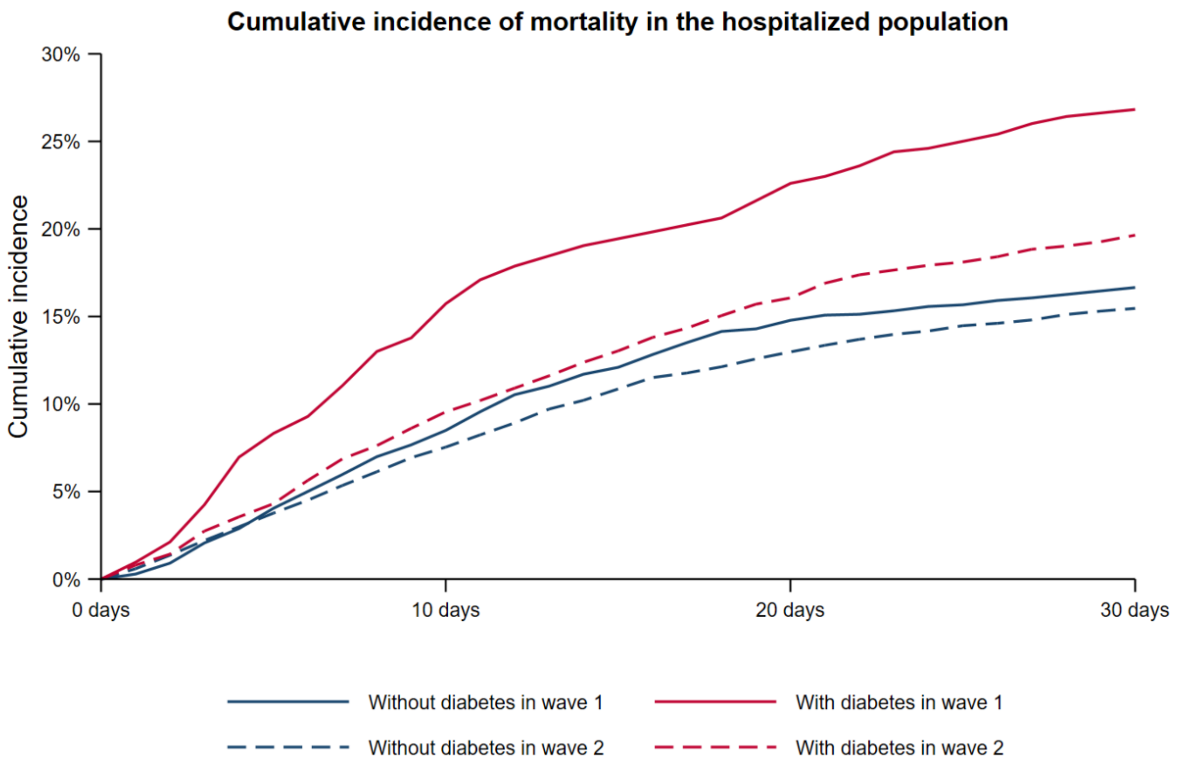

ESM Figure 3B. COVID-19 related mortality stratified by age and sex in the hospitalized population during the first and second wave of COVID-19 in Denmark according to diabetes status. Time was defined according to the start date of hospitalization.

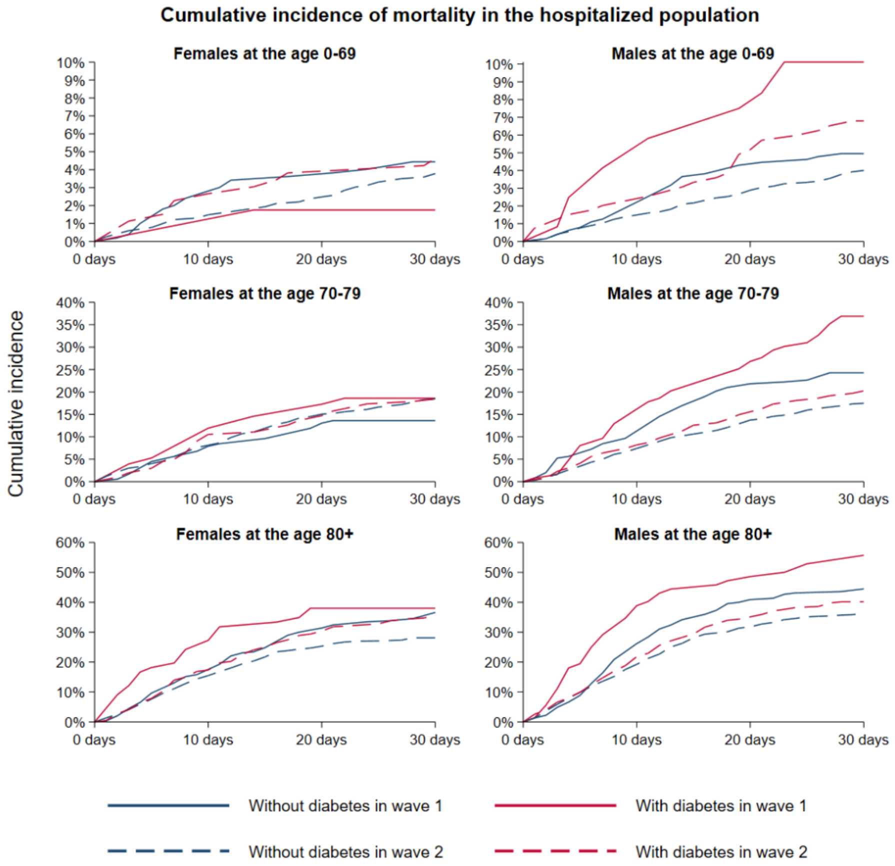

ESM Figure 4A. Crude cumulative incidence of COVID-19 related admission to intensive care unit (ICU) during the first and second wave of COVID-19 in Denmark according to diabetes status. Time was defined according to the start date of the two waves.

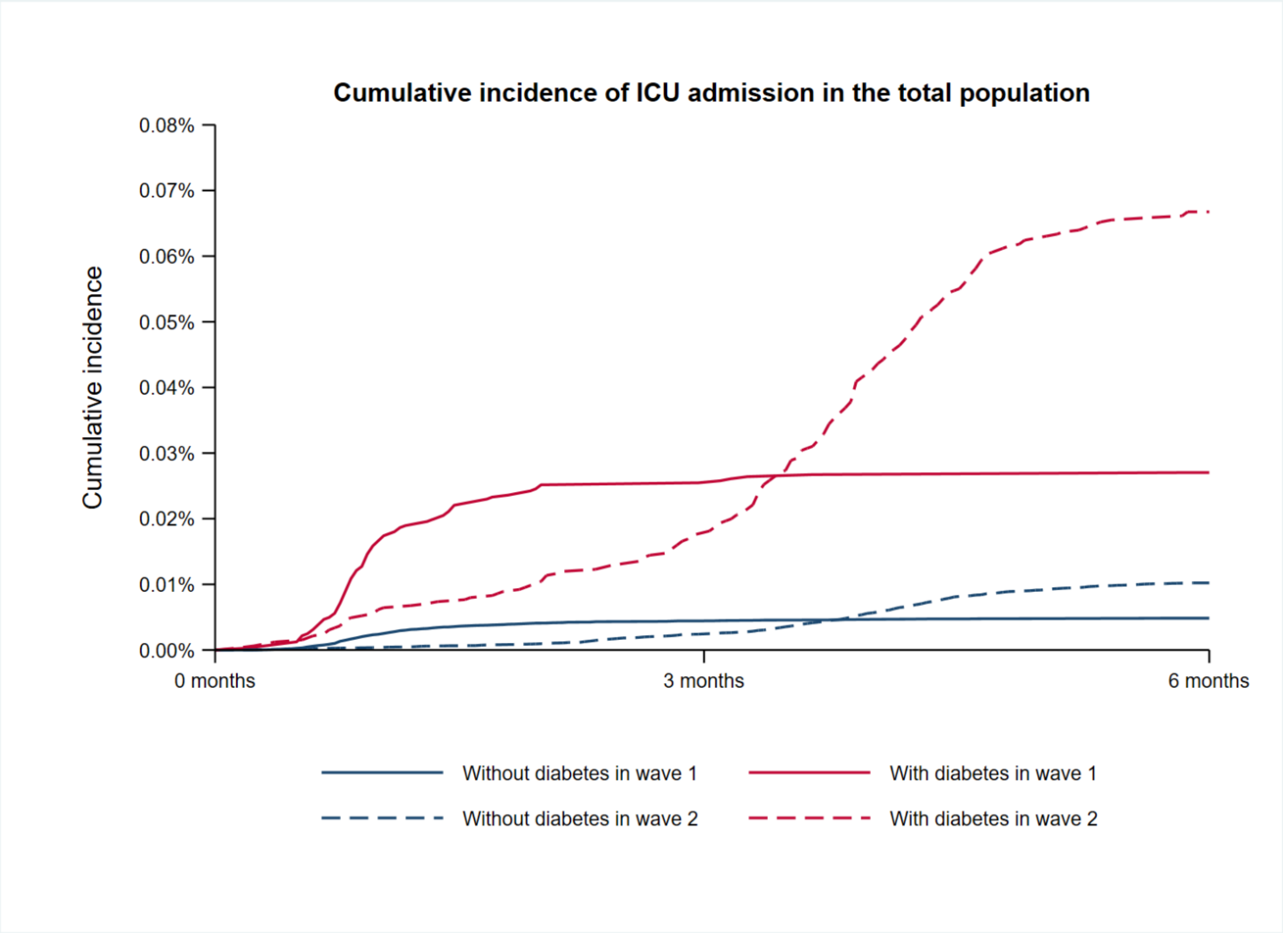

ESM Figure 4B. Cumulative incidence, stratified by age and sex, of COVID-19 related admission to intensive care unit (ICU) during the first and second wave of COVID-19 in Denmark according to diabetes status. Time was defined according to the start date of the two waves.

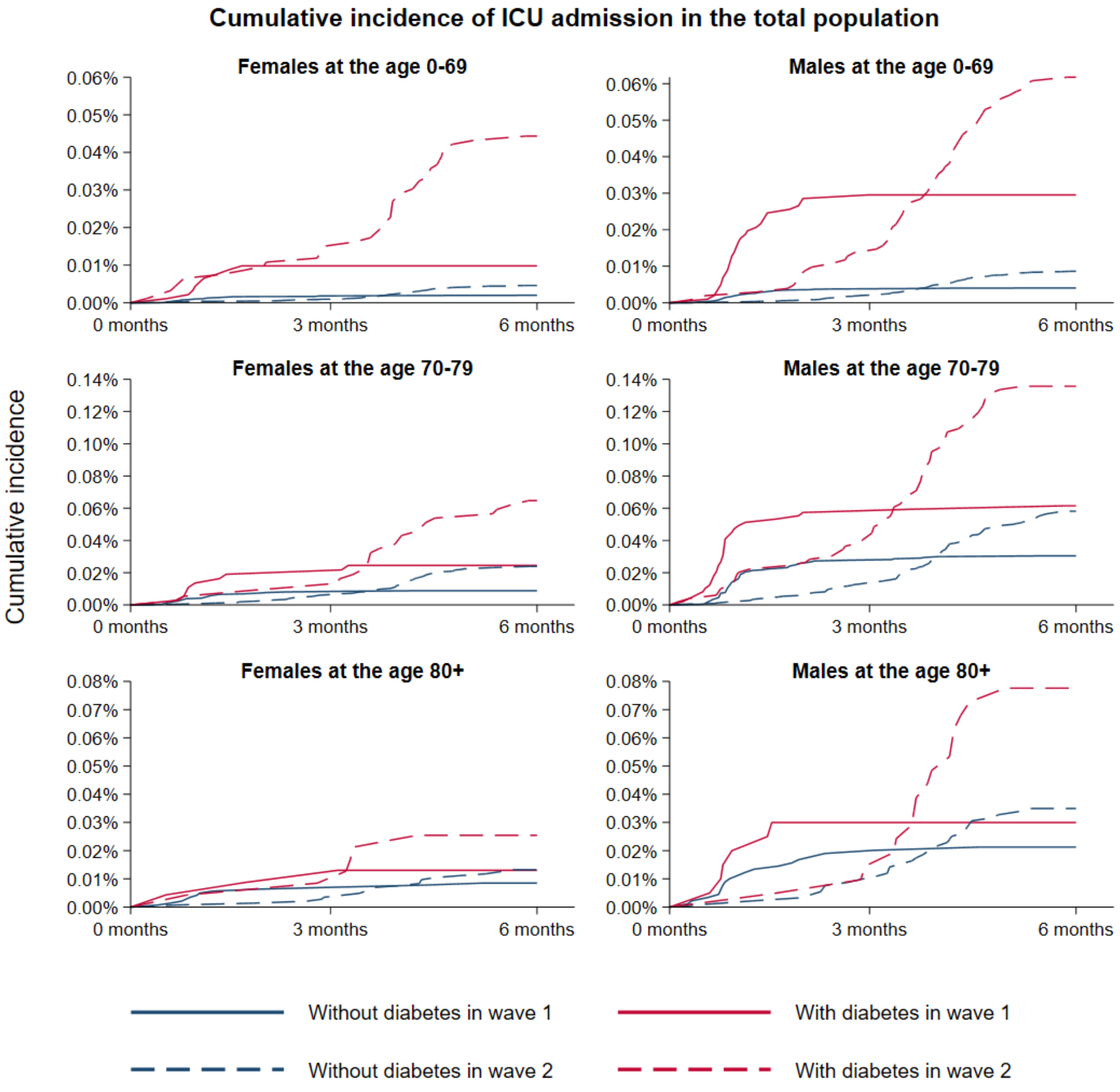

ESM Figure 5A. Crude cumulative incidence of COVID-19 related admission to intensive care unit (ICU) in the hospitalized population during the first and second wave of COVID-19 in Denmark according to diabetes status. Time was defined according to the start date of hospitalization.

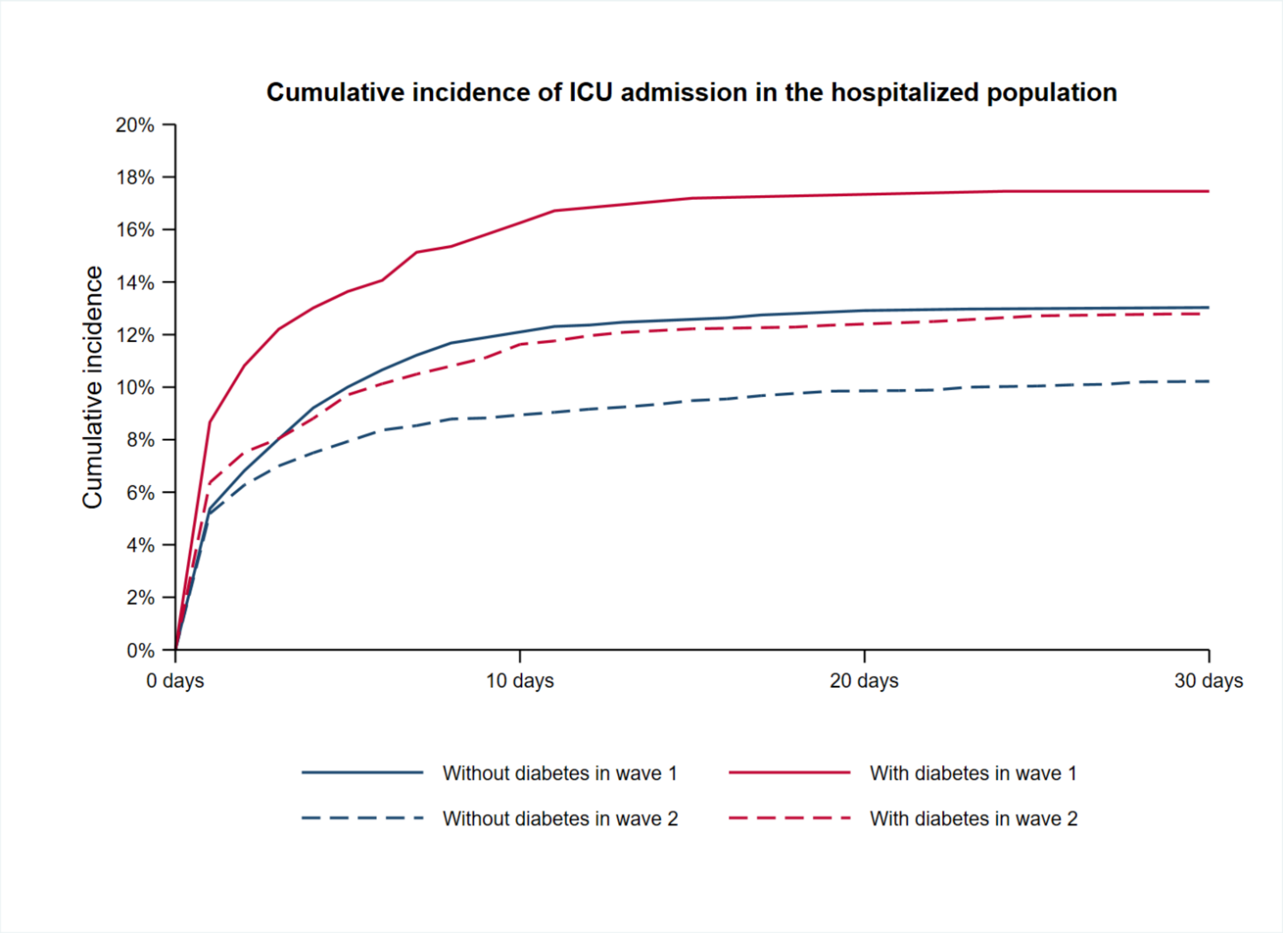

ESM Figure 5B. Cumulative incidence, stratified by age and sex, of COVID-19 related admission to intensive care unit (ICU) in the hospitalized population during the first and second wave of COVID-19 in Denmark according to diabetes status. Time was defined according to the start date of hospitalization.

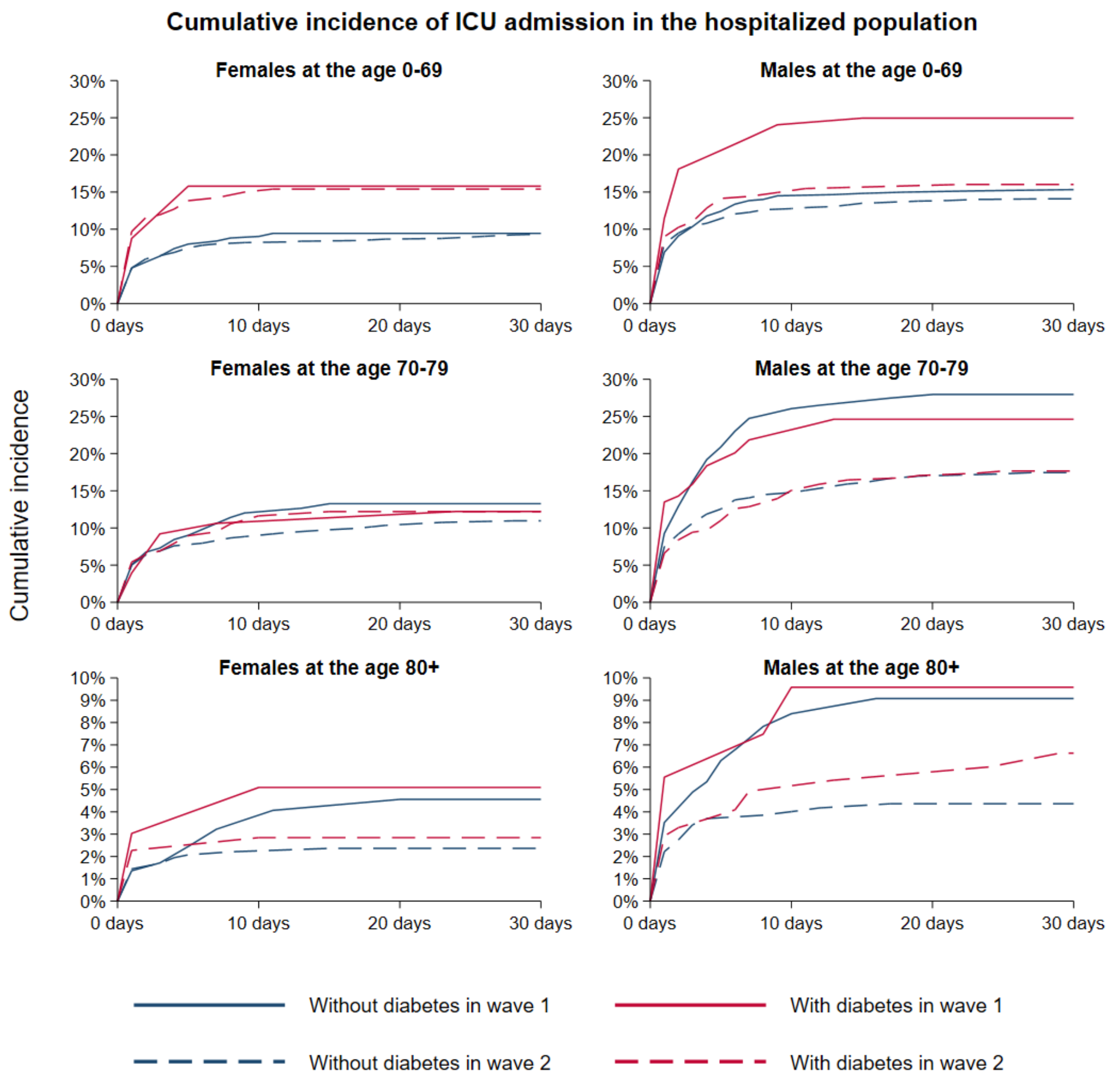

Supplement: Supplementary file 1 [file Presentation_1.pdf]
